# Seven-octave ultrabroadband metamaterial absorbers via quality-factor-weighted mode density modulation

**DOI:** 10.1093/nsr/nwaf199

**Published:** 2025-05-20

**Authors:** Nengyin Wang, Sibo Huang, Zhiling Zhou, Din Ping Tsai, Jie Zhu, Yong Li

**Affiliations:** Institute of Acoustics, Tongji University, Shanghai 200092, China; Department of Electrical Engineering, City University of Hong Kong, Hong Kong, China; Institute of Acoustics, Tongji University, Shanghai 200092, China; Department of Electrical Engineering, City University of Hong Kong, Hong Kong, China; Institute of Acoustics, Tongji University, Shanghai 200092, China; Institute of Acoustics, Tongji University, Shanghai 200092, China

**Keywords:** ultrabroadband absorption, metamaterial, multiple resonant modes, quality-factor-weighted mode density

## Abstract

Absorption is a crucial parameter in shaping wave propagation dynamics, yet achieving ultrabroadband absorption remains highly challenging, particularly in balancing a low-frequency and broad bandwidth. Here, we present a metamaterial absorber (MMA) capable of achieving simultaneous spectral coverage across a seven-octave range of near-perfect absorption from 100 to 12 800 Hz by engineering the quality-factor-weighted (*Q*-weighted) mode density. The *Q*-weighted mode density considers mode density, resonant frequencies, radiative loss and intrinsic loss of multiple resonant modes, providing a comprehensive approach to governing broadband absorption properties. By optimizing the number of resonant modes and managing intrinsic losses, our approach achieves an intensive *Q*-weighted mode density across an ultrawide bandwidth, enabling ultrabroadband absorption with high efficiency. These findings significantly advance the bandwidth capabilities of state-of-the-art MMAs and pave the way for the development of ultrabroadband metamaterial devices across various wave systems.

## Teaser

A novel meta-absorber efficiently captures noise across seven octaves–spanning most of the human hearing range–by engineering how fine-tuned resonator arrays manipulate and dissipate energy.

## INTRODUCTION

Absorption is an essential physical phenomenon where wave energy is transformed into other types within a medium or on its interfaces. It plays a crucial role in determining the propagation dynamics of sound waves, electromagnetic waves and beyond. For instance, by judiciously modulating the absorption in non-Hermitian systems, the accompanying absorption can support effective wave manipulation and induce exotic phenomena such as coherent perfect absorption [1,2], asymmetric transmission [3,4] and reflectionless scattering modes [5,6].

High-efficiency broadband absorption, in particular, has been a longstanding pursuit due to its significant scientific value and engineering potential [7–16]. Conventional non-resonant resistive materials exhibit fine performance at the cost of a bulky volume, while the absorption properties lack tunability [12,14,17]. Recently, metamaterial absorbers (MMAs) have demonstrated exceptional wave manipulation capabilities for customized absorption characteristics by harnessing the resonances of subwavelength structures, which provide more degrees of tuning freedom [18–22]. However, the dispersive nature of resonances strongly restricts the operating bandwidth [4,23–25], confining the highly efficient absorption to narrow-band ranges [26–33].

To make breakthroughs in the bandwidth, various physical mechanisms have been investigated. The most common design strategy is based on the mode density by coupling as many local resonances as possible. In this case, a wide operating bandwidth heavily requires a substantial number of resonant modes [7,12,16,34–36]. Governed by this mechanism, the cutting-edge theoretical and experimental achievements of high-efficiency absorption bandwidths for MMAs are less than 30, to the best of our knowledge [7,9,13,14,16,37–43]. Here, the ratio between the upper and lower bounds of the operating band is used as a scale to evaluate the bandwidth. Additionally, the impact of complex higher-order propagating modes becomes significant in high-frequency regimes, imposing barriers to theoretical design and practical implementation. So far, achieving ultrabroadband high-efficiency MMAs over a hundred-fold bandwidth remains elusive.

In this study, we theoretically and experimentally demonstrate a seven-octave (128-fold-bandwidth) MMA. We introduce the quality-factor-weighted (*Q*-weighted) mode density as a fundamental concept to facilitate the achievement of ultrabroadband MMAs. The *Q*-weighted mode density incorporates the mode density, the resonant frequencies, radiative loss and intrinsic loss of multiple resonant modes (MRMs), manifesting a more fundamental and explicit connection with broadband absorption properties than mode density alone. It provides an alternative new pathway to develop ultrabroadband high-efficiency MMAs by modulating the *Q* factors of a relatively small number of resonant modes, lifting the requirement of a huge number of resonant modes that are considerably challenging for MMAs in various wave systems. To demonstrate this, we introduce an acoustic system consisting of an array of cascade-parallel Helmholtz resonators and a covering wire mesh with micrometer-scale thickness. The resonator array provides a moderate mode density and the wire mesh enhances the *Q*-weighted mode density. Furthermore, we establish a theoretical framework and an experimental method to enable the design and measurement of ultrabroadband MMAs involving the consideration of high-order radiating waves. Finally, the presented MMA achieves high-efficiency absorption over 100–12 800 Hz with an average absorption coefficient of 0.944 and a thickness of 35.3 cm, approaching the causality-governed minimal thickness [9,16,44].

## RESULTS AND DISCUSSION

### Modulation based on the *Q*-weighted mode density

**Figure 1.**
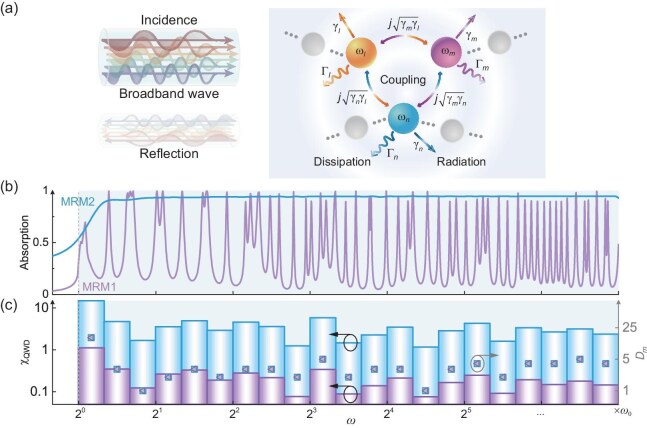
The physical mechanism of ultrabroadband MMAs based on MRM systems. (a) Schematic of the mode interactions of MRM systems. The three spheres with different colors represent three different modes. The gray spheres indicate the other different modes. We denote by ${\omega }$, ${\gamma }$ and ${\Gamma }$ the resonant angular frequency, the radiative loss and intrinsic loss, respectively. Subscripts *m, n* and *l* denote different modes; $j\sqrt{{\gamma _l}{\gamma _m}}$ indicates the coupling between mode *l* and mode *n*, as illustrated by the bi-directional arrows. The indications of other mode couplings follow a similar representation. (b) Absorption spectra of MRM1 (purple line) and MRM2 (blue line). (c) The *Q*-weighted mode density (bar graphs, corresponding to the left axis) and mode density (purple triangle and blue square scatter plots, corresponding to the right axis) of MRM1 (purple) and MRM2 (blue).

MRM systems offer exceptional potential for broadband wave control, thereby facilitating the achievement of ultrabroadband absorption [35]. Through tuning the essential qualities of each resonant mode (resonant frequency, radiative loss and intrinsic loss) and the coupling among the modes [Fig. 1(a)], MRM systems can effectively suppress the dispersion of resonant mode, thereby facilitating broadband absorption. Previous studies demonstrate that a high mode density within the target band is required for broadband absorption [7,16,35,36]. The mode density is defined as $D_{{m}} = N / \log _2(\omega _{\max }/\omega _{\min })$, where *N* is the number of resonant modes within the target frequency regime between ${\omega _{\max }}$ and ${\omega _{\min }}$. Accordingly, achieving large $D_{{m}}$ within a broader frequency regime demands a greater number of resonant modes, while when it comes to an ultrabroadband frequency regime, it becomes rather challenging. Besides, due to the complex interactions among the modes within an ultrawide bandwidth, only achieving a high mode density cannot always ensure high-efficiency absorption. To overcome these challenges, we further take the other important qualities of resonant modes into consideration and present the concept of the *Q*-weighted mode density. The quality (*Q*) factor can be expressed as $Q_n = \omega _n /[2( \gamma _n + \Gamma _n)]$, where *n* denotes the *n*th mode, and $\omega _n$, $\gamma _n$ and $\Gamma _n$ respectively represent the resonant angular frequency, radiative loss and intrinsic loss. In this study, the introduced *Q*-weighted mode density (${\chi _{{\rm {QMD}}}}$) is given by


(1) \begin{eqnarray*}
\chi _{\rm {QMD}} = \frac{\sum _{n = 1}^N Q_n^{-1}}{\log _2(\omega _{\max }/\omega _{\min })},
\end{eqnarray*}

which encompasses the mode density, the *Q* factors and the interactions of MRMs.

To demonstrate the design concept based on the *Q*-weighted mode density over the mode density, we employ coupled mode theory (see the section entitled ‘Coupled-mode theory for the MRM’ below) to calculate the absorption coefficient of two MRM systems with the same mode density within the same frequency band, where the absorption spectra are compared in Fig. 1(b), Oscillations and numerous dips in the absorption spectrum can be observed for MRM1. In contrast, MRM2 exhibits significantly superior broadband absorption performance over MRM1 owing to the better modulation of the resonant modes’ essential qualities and the improved interactions among the modes. Therefore, although the two systems possess identical $D_{{m}}$ [indicated by purple triangles and blue squares in Fig. 1(c)], the substantial differences in absorption spectra can arise from the different ${\chi _{{\rm {QMD}}}}$, where the MRM system with larger ${\chi _{{\rm {QMD}}}}$ (for MRM2) is capable of more enhanced broadband absorption performance than that with smaller ${\chi _{{\rm {QMD}}}}$ (for MRM1), as depicted by the purple and blue bars in Fig. 1(c). Consequently, by embracing the *Q* factor, ${\chi _{{\rm {QMD}}}}$ fundamentally and comprehensively interprets the physical mechanisms and effect of mode couplings (see Note S1 for details), leading to a more effective physical parameter for characterizing and designing the absorption properties of ultrabroadband MMAs based on MRM systems than $D_{{m}}$. Specifically, to achieve flat and high-efficiency absorption across multiple octaves [Fig. 1(b), blue curve], the modulation of $\chi _{\rm {QMD}}$ follows a generalized workflow. First, the target absorption spectrum and structural constraints (e.g. resonator number) are defined. Second, $\chi _{\rm {QMD}}$ is optimized by maximizing its value across the target band to sustain high absorption efficiency, with prioritized higher $\chi _{\rm {QMD}}$ at the starting frequencies to ensure a steep absorption rise (critical for compact designs [7]). Finally, all parameters are optimized via the genetic algorithm, under the balance of the design target and physical limit (see Note S3D for the detailed workflow on designing absorbers).

### Fundamental approaches for modulating broadband absorption performance

**Figure 2.**
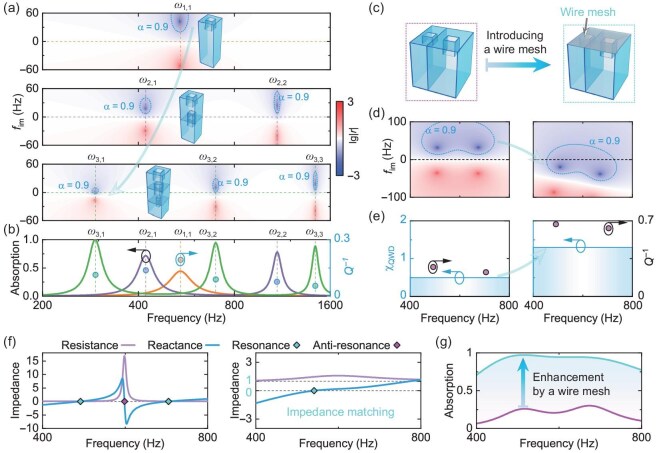
Approaches for enhancing ${\chi _{{\rm {QMD}}}}$ in the acoustic system. (a) Acoustic frequency response of cascade structures. The complex-frequency response is described by the logarithm of the reflection coefficient amplitude [$\lg ({|r|})$], corresponding to single-layer (top), double-layer (middle) and three-layer (bottom) MCNEHRs. Insets show the structures. Here ${f_{{\rm {im}}}}$ represents the imaginary part of the complex frequency, corresponding to loss; ${f_{{\rm {im}}}} > 0\ ( { <\! 0})$ indicates the increase (decrease) in loss. The frequencies corresponding to reflection zeros are the resonance frequencies ${\omega _{l,n}}\,( {l = 1,2,3;n = 1,2,3} )$. We denote by $\alpha =1 - {|r|^2}$ the absorption coefficient. (b) Acoustic absorption spectra (solid lines, left axis) and ${Q^{ - 1}}$ at each resonance mode (circles, right axis) for corresponding MCNEHRs, where the curves and circles correspond to the real axes of the complex planes in (a) with matching colors. (c–g) Vertical division of the parallel structure and the change in the MMA performance by introducing a wire mesh. (c) Illustration of the parallel structure (left) and the structure covered with a wire mesh (right). (d) Complex frequency response of $\lg ( {| r |} )$ corresponding to (c). (e) Distributions of ${Q^{ - 1}}$ (magenta circles, right axis) and ${\chi _{{\rm {QMD}}}}$ (blue bars with shading, left axis) corresponding to (c). (f) Acoustic impedance before (left) and after (right) introducing a wire mesh. Rhombuses indicate resonance (cyan) and antiresonance (magenta) locations. (g) Acoustic absorption spectra corresponding to structures without (magenta) and with (cyan) the wire mesh.

The characteristics of resonance are similar across various wave systems, which makes the above design concept general for various wave systems. In the following, we demonstrate the concepts based on acoustic systems. Firstly, we need to construct acoustic resonators that can support an adequate number of resonant modes. Meanwhile, these resonators are anticipated to allow for high-degree-of-freedom modulations of the mode properties for achieving highly tunable mode couplings. In acoustics, Helmholtz resonators (HRs) are a classic implementation for introducing resonances [24]. To obtain high-degree-of-freedom modulations of the mode properties, we propose the multilayer cascade neck-embedded Helmholtz resonator (MCNEHR) by transversely dividing the cavity into different sections using intermediate plates with embedded necks, based on the HR [7]. The radiative and intrinsic loss of the resonant modes can be adjusted via the embedded necks (see Note S2A for details). The MCNEHRs facilitate the resonators to have resonant frequencies in the low-frequency regime and increase the number of modes [Fig. 2(a); see also Fig. S3], promoting high-efficiency absorption in the low-frequency regime with sub-wavelength structures (the geometry parameters are detailed in Table S1). However, for a single MCNEHR, the supported modes are typically uncoupled, and $Q^{-1}$ of the modes tends to decrease with the number of layers [circles in Fig. 2(b)]. This characteristic of single MCNEHRs hinders effective manipulation of the ${\chi _{{\rm {QMD}}}}$, resulting in relatively narrow absorption bandwidths [see the absorption spectra in Fig. 2(b)].

In addition, vertical spatial division [left panel of Fig. 2(c)] can also increase the mode density, and, more importantly, introduce radiation coupling among different component resonators (the geometry parameters are detailed in Table S2). Radiation coupling plays a crucial role in suppressing the dispersion nature of resonances [left panel of Fig. 2(d)]. However, it is difficult for resonators to have sufficient and controlled intrinsic loss across a wide bandwidth. When the intrinsic loss of the resonators is insufficient, $Q^{-1}$ and ${\chi _{{\rm {QMD}}}}$ will be inadequate [left panel of Fig. 2(e)]. In this case, the quasi-perfect absorption region (absorption efficiency exceeding 90%) will not fall on the real axis [left panel of Fig. 2(d)], hindering the realization of broadband absorption. Furthermore, the presence of reflection zero points above the real axis indicates that the absorber includes redundant thickness (see Note S6 for details). Hence, although the transverse and vertical spatial divisions of HRs can effectively generate more resonant modes, sufficient intrinsic loss is required for optimal broadband absorption performance. Here, we present a simple approach to effectively modulate the intrinsic loss of the resonators across an extensive bandwidth by covering their surfaces with an extremely thin wire mesh [right panel of Fig. 2(c); see the Methods section below and Notes S2A and S4A for details]. The additional intrinsic loss offered by the wire mesh can be adjusted by the mesh count ($C_{{m}}$), facilitating the significantly enhanced $Q^{-1}$ and ${\chi _{{\rm {QMD}}}}$ [right panel of Fig. 2(e)] and improved broadband performance [right panel of Fig. 2(d), where the quasi-perfect absorption region intersects the real axis]. Moreover, the reflection zeros are shifted to the lower half-plane, ensuring that the absorber achieves the minimum thickness determined by the causality constraint.

The dispersive features of the resonators are evidently suppressed by adjusting the intrinsic loss supported by the wire mesh [Fig. 2(f)]. Without the aid of a wire mesh, the resistance (real part of impedance) and reactance (imaginary part of impedance) vary dramatically around the resonant frequency, exhibiting strong dispersion [left panel of Fig. 2(f)]. The introduction of a wire mesh restricts the resistance and reactance within a low fluctuation range, enabling the MMA to accomplish broadband impedance matching, with the acoustic resistance nearly reaching 1 and the acoustic reactance approaching 0 [right panel of Fig. 2(f)]. Meanwhile, the suppression of anti-resonance is achieved. Consequently, the absorber’s absorption performance is significantly improved in terms of both the bandwidth and the absorption coefficients [Fig. 2(g)].

### Performance of the seven-octave MMA

**Figure 3.**
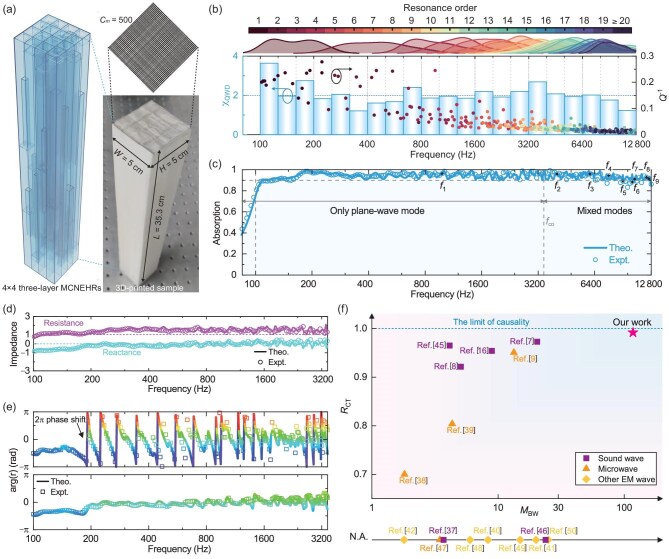
Seven-octave metamaterial absorber. (a) Schematic of the seven-octave MMA, consisting of a 500-mesh wire mesh (upper right) covering the top surfaces of 16 parallel three-layer MCNEHRs (left), with a 3D printed sample shown in the right panel. (b) The ${\chi _{{\rm {QMD}}}}$ (bar charts) within each 1/3 octave and the theoretical $Q^{-1}$ for resonant modes (colored dots). The distribution of different orders of resonant modes (color-filled curves). The absorption peak less than 0.1 is ignored. (c) Theoretical (curves) and experimental (circles) absorption spectra of the MMA. The labels $f_1$ to $f_9$ are points in frequency bands with different numbers of propagating modes. (d) Theoretical (curves) and experimental (circles) acoustic resistance and reactance spectra below ${f_{\rm {co}}}$. (e) Theoretical (curves) and experimental (squares) reflection phases of the plane-wave modes corresponding to the bare MMA (top) and the MMA covered with a wire mesh (bottom). (f) Performance comparison of absorbers in different wave systems. Here, N.A. indicates that $R_{\rm {CT}}$ is not available in the relevant work.

Guided by the aforementioned design strategies (see Fig. S8 for the specific workflow), we constructed an MMA employing only 16 three-layer MCNEHRs covered with a layer of wire mesh, with overall width $W = 5$ cm, height $H = 5$ cm (cutoff frequency ${f_{\rm {co}}} = 3432$ Hz, defined in Note S9B) and thickness $L = 35.3$ cm [Fig. 3(a)]. Based on the developed theory (see Notes S3A and S3B), we performed global optimization to enhance the *Q*-weighted mode density and achieve extremely broadband high-efficiency acoustic absorption. The distribution of $Q^{-1}$ demonstrates that the MMA could generate multiple effective resonant modes over an extensive broadband range spanning seven octaves from 100 to 12 800 Hz [color-filled circles in Fig. 3(b)]. After introducing the wire mesh, ${\chi _{{\rm {QMD}}}}$ is significantly enhanced [blue bars in Fig. 3(b) and in Fig. S13(a)]. Such a ${\chi _{{\rm {QMD}}}}$ distribution leads to extremely broadband high-efficiency absorption spanning seven octaves, exhibiting an average absorption coefficient of 0.944 (0.941 in the experiment) from 100 to 12 800 Hz [Fig. 3(c)]. Specifically, within 109–12 800 Hz, the presented MMA realizes quasi-perfect absorption. The experimental and theoretical results are in good agreement, except for a slight difference in the high-frequency regime, which may be caused by the three-dimensional (3D) printing imperfections. Furthermore, within the frequency regime below the waveguide’s cut-off frequency ($f < {f_{{\rm {co}}}}$, where only the plane-wave mode exists), the MMA exhibits perfect impedance matching and ideal over-damping characteristics [Fig. 3(d)]. Moreover, our presented MMA exhibited nearly perfect absorption performance in the frequency regime above ${f_{{\rm {co}}}}$ ($f > {f_{{\rm {co}}}}$, where both plane-wave and higher-order modes propagate), as will be shown later. Under varying oblique incident angles, the MMA can also provide excellent absorption performance, indicating the robustness of the MMA’s acoustic absorption performance and its insensitivity to incident angles (see Fig. S16).

From the results above, it can be observed that the wire mesh has a significant impact on the overall absorption performance of the MMA despite its thin thickness of only 0.05 mm. Compared with the bare MMA (see Note S4C for details), the addition of the wire mesh remarkably improves the intrinsic loss and the half-maximum relative bandwidth (i.e. $Q^{-1}$) of the MRM. This enhancement leads to increased ${\chi _{{\rm {QMD}}}}$, particularly in the low-frequency regime, effectively suppressing absorption dips caused by anti-resonances and impedance oscillations [Fig. 3(d); see also Fig. S13(b)]. The effect of the wire mesh can also be understood from the perspective of the phases of reflection coefficients. Without the wire mesh, numerous phase shifts of 0 to $2\pi$ can be observed [top panel of Fig. 3(e)]. This phenomenon arises from the condition of the reflective zeros on the upper-half complex frequency plane (see Fig. S15). In contrast, the MMA with a wire mesh exhibits the minimum phase shift frequency dependence [44] throughout the band [bottom panel of Fig. 3(e)]. This result reveals that the zeros and poles reside on the same side of the real axis, indicating that the absorber has reached ${L_{\min }}$. Furthermore, we plotted the Riemann surface of the reflection coefficients of the plane-wave modes over the entire target band and the reflection phases on their real axes, which further verifies this point (see Note S4C for details).

To better evaluate the performance of the presented MMA, we introduce two quantitative indicators, the bandwidth multiple (${M_{{\rm {BW}}}}$) and the causality thickness ratio (${R_{{\rm {CT}}}}$), for comprehensively comparing the absorbers in various wave systems. The bandwidth multiple ${M_{{\rm {BW}}}}$ is defined as ${M_{{\rm {BW}}}} = {f_{\max }}/{f_{\min }}$, where ${f_{\max }}$ and ${f_{\min }}$ respectively represent the maximum and minimum frequencies of the frequency band consistently exhibiting quasi-perfect absorption. For the MMA presented in this work, the quasi-perfect absorption band ranges from 109 to 12 800 Hz, resulting in ${M_{{\rm {BW}}}} = 117$. Besides, ${R_{{\rm {CT}}}}$ is defined as $R_{\rm {CT}} = L_{\rm {min}}/ L$, where *L* represents the sample thickness. For our presented MMA, $L = 35.3$ cm and ${L_{\min }}=35$ cm (see Note S6A for the retrieval formula), and thus ${R_{{\rm {CT}}}}{{ = 0.991}}$. We emphasize that ${R_{{\rm {CT}}}}$ can only reach the maximum value 1 limited by the causal constraint when $L = {L_{\min }}$, i.e. the thickness of the absorber reaches the minimum thickness of the causal domination [blue dashed line in Fig. 3(f)]. Therefore, ${R_{{\rm {CT}}}}$ precisely highlights the optimal trade-off between absorption properties and the thickness of MMAs. Furthermore, ${M_{{\rm {BW}}}}$ carries the importance of the effective bandwidth. These two indicators enable a comprehensive evaluation of the absorber’s overall performance. Compared to other absorbers of various wave systems [Fig. 3(f); see also Note S8 for details] [7–9,16,37–42,45–50], our presented MMA boasts an order of magnitude higher ${M_{{\rm {BW}}}}$ and mostly reaches the physical limit of ${R_{{\rm {CT}}}}$.

### Absorption performance under mixed-mode incidence

**Figure 4.**
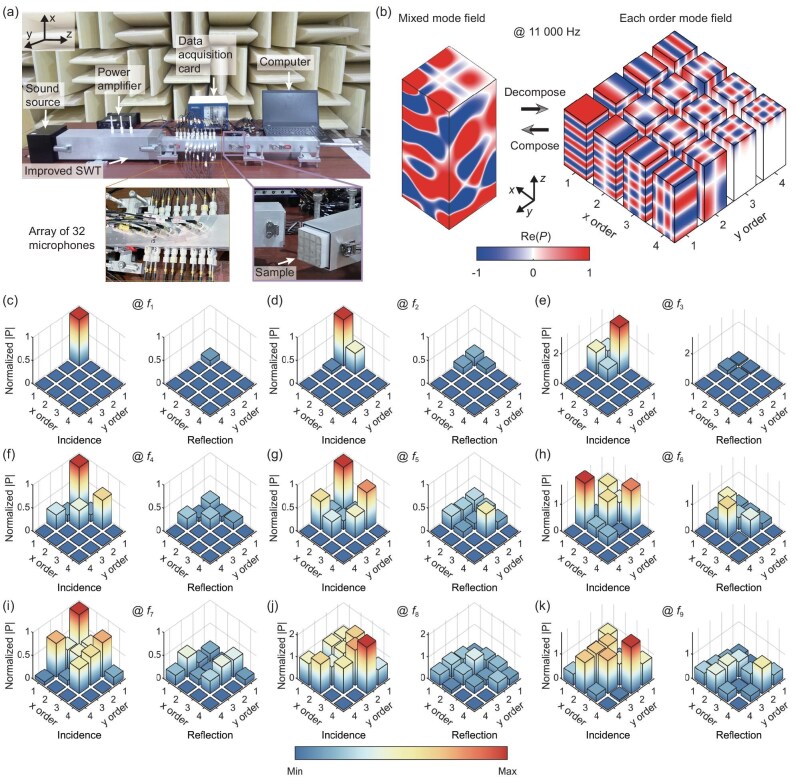
Absorption performance of the MMA for mixed modes. (a) Schematic of the experimental platform in the anechoic chamber. Thirty-two microphones are placed in the measurement section, supporting mode decomposition of the highest order of 16. (b) Compose and decompose incident fields at 11 000 Hz consisting of simultaneous plane-wave modes (only one), high-order propagation modes (12 in total) and evanescent wave modes (three in total). (c)–(k) Incident and reflected mode amplitudes from experimental results corresponding to frequency points $f_1$ to $f_9$ (with 1, 3, 4, 6, 8, 9, 11, 13 and 15 propagation modes, respectively) in the frequency bands with different numbers of propagation modes in Fig. 3(c).

Because of to the inadequacies in previous theories and challenges in practical implementation, most research on acoustic MMAs has steered clear of investigating mixed-mode absorption properties, significantly hindering the advancement of ultrabroadband acoustic MMAs. To overcome this bottleneck, a more robust theoretical design framework and a more comprehensive experimental testing platform [Fig. 4(a); the multi-mode standing-wave tube (SWT)] have been developed. The platform employs 32 microphones to decompose the mixed modes above ${f_{\rm {co}}}$, enabling the calculation of the absorption coefficients for higher-order propagating modes (see the section entitled ‘Experimental setup’ below and Note S9A for details). In addition, our developed theory enables accurate calculation of the absorption performances of MMAs beyond the ${f_{\rm {co}}}$ limitation. As a result, the experimental results fit well with the theoretical predictions [Fig. 3(c)].

The propagating modes at different frequencies are determined by the dispersion relation (see Note S9B). For instance, at 11 000 Hz [Fig. 4(b)], the plane-wave modes of order (1,1) and 12 higher-order modes ranging from orders (1,2) to (3,3) exist. The remaining higher-order modes are all evanescent modes, as illustrated by orders (3,4), (4,3) and (4,4) in Fig. 4(b). Based on this classification, the entire operational bandwidth can be divided into nine sub-bands with varying numbers of propagating modes (see Fig. S22). The overall absorption performance of the mixed modes is demonstrated in Fig. 3(c) (above ${f_{\rm {co}}}$). Furthermore, to provide a clearer illustration of the absorption of the mixed modes, a frequency point was selected from each of the nine frequency bands (${f_{1}}$ to ${f_{9}}$) and decomposed using the aforementioned measurements. Panels (c)–(k) of Fig. 4 display the measured incident (left panel) and reflected (right panel) mode amplitudes for each order of the propagating modes from ${f_{1}}$ to ${f_{9}}$ (see Fig. S23 for the mode energy flow). These figure panels clearly demonstrate the highly robust absorption performance of our presented MMA under the incidences of different mixed modes. Nearly perfect absorption is observed for the majority of modes at various frequencies, with only a trivial energy transformation occurring between different modes (see Fig. S24 for the mode transformation diagrams). This energy transformation results in a small number of higher-order modes exhibiting a reflection amplitude slightly larger than the incidence amplitude, such as the (1,2) mode in ${f_{2}}$ [Fig. 4(d)] and the (3,2) mode in ${f_{6}}$ [Fig. 4(h)]. However, these modes only pose negligible influence on the overall absorption performance, because the plane-wave modes [(1,1) in Fig. 4(c)–(k)] with the highest energy share are significantly absorbed.

## CONCLUSION

In conclusion, our MMA achieves ultrabroadband absorption across an unprecedented seven-octave range with remarkable efficiency. By leveraging the quality-factor-weighted mode density to optimize resonant modes and manage intrinsic losses, we enable effective absorption from 100 to 12 800 Hz. This innovative approach not only advances the performance of metamaterial absorbers, but also establishes a robust framework for extending ultrabroadband applications to diverse wave systems, including non-Hermitian systems and novel topological devices. Moreover, the methodology shows significant potential for new applications, such as in aerospace engines, where ultrabroadband absorption could greatly enhance performance and efficiency.

## METHODS

### Coupled-mode theory for the MRM

As shown in Fig. 1(a), a resonant system with *N* coherently coupled modes has mode amplitudes $\tilde{A} = {( {{{\tilde{a}}_1}, \dots ,{{\tilde{a}}_n}, \dots ,{{\tilde{a}}_N}} )^\top }$, whose temporal evolution can be described by coupled-mode theory as


(2) \begin{eqnarray*}
- j\frac{d}{{dt}}\tilde{A} = H\tilde{A} + \tilde{b},
\end{eqnarray*}

where ${\tilde{a}_n} = {a_n}{e^{j\omega t}}$ represents the amplitude of the *n*th resonant mode, *j* is the unit imaginary number, *H* is the Hamiltonian of these *N* modes, i.e.


(3) \begin{eqnarray*}
H =\left({\begin{array}{c@{}c@{}c@{}c@{}c} {\Omega _1}& \cdots &{j\sqrt{{\gamma _1}{\gamma _n}} }& \cdots &{j\sqrt{{\gamma _1}{\gamma _N}} } \\
\vdots & \ddots & \vdots & \vdots & \vdots \\
{j\sqrt{{\gamma _1}{\gamma _n}} }& \cdots &{{\Omega _n}}& \cdots &{j\sqrt{{\gamma _n}{\gamma _N}} }\\
\vdots & \vdots & \vdots & \ddots & \vdots \\
{j\sqrt{{\gamma _1}{\gamma _N}} }& \cdots &{j\sqrt{{\gamma _n}{\gamma _N}} }& \cdots &{{\Omega _N}} \end{array}}\right)
\end{eqnarray*}

and $\tilde{b} = {( {\sqrt{{\gamma _1}} {{\tilde{S}}_{\rm {i}}}, \dots ,\sqrt{{\gamma _n}} {{\tilde{S}}_{\rm {i}}}, \dots , \sqrt{{\gamma _N}} {{\tilde{S}}_{\rm {i}}}} )^\top }$, with ${\Omega _n} = {\omega _n} + j{\gamma _n} + j{\Gamma _n}$, ${\omega _n}$ the resonance frequency of the *n*th mode, ${\gamma _n}$ the corresponding radiative decay rate (i.e. radiative loss, which is the amount of energy lost by wave radiation from the resonant structure into the surrounding medium; this type of loss is mainly determined by the geometry of the structure and the coupling between the resonant modes and free space) and ${\Gamma _n}$ the dissipative decay rate (i.e. intrinsic loss, which is related to material dissipation within the structure, such as thermal loss, viscous loss or resistive loss; this type of loss is independent of external radiation and is instead determined by the intrinsic material properties). For the source term ${\tilde{b}}$, ${\tilde{S}_{\rm {i}}} = {S_{\rm {i}}}{e^{j\omega t}}$ describes the incident wave, while $\sqrt{{\gamma _n}}$ quantifies the coupling strength between the source and the *n*th mode. The non-diagonal terms of *H* describe the interaction between modes, i.e. the coupling effect. The coupling term $\sqrt{{\gamma _n}{\gamma _m}}$ ensures energy conservation, with *j* preserving time-reversal symmetry [51,52].

Eliminating the time factor term ${e^{j\omega t}}$ gives $( {I\omega - H} )A = b$, where *I* is the unit matrix, and solving the system of linear non-homogeneous equations gives [15,53]


(4) \begin{eqnarray*}
{a_n} &=& \frac{{j{S_{\rm {i}}}}}{{1 + \sum _m {{[{{\gamma _m}}}/{{(j\omega - j{\omega _m} + {\Gamma _m})]}}} }} \\
&& \times \, \frac{{\sqrt{{\gamma _n}} }}{{j\omega - j{\omega _n} + {\Gamma _n}}}.
\end{eqnarray*}

Furthermore, the reflection coefficient of the coupled system, *r*, can be expressed as


(5) \begin{eqnarray*}
r = 1 + 2j\sum _m {\sqrt{{\gamma _m}} \frac{{{a_m}}}{{{S_{\rm {i}}}}}} .
\end{eqnarray*}

Substituting (5) into the impedance expression $Z = ( 1 + r ) / (1 - r)$ yields


(6) \begin{eqnarray*}
Z &=& \frac{1}{{\sum _m {{\lbrace {{\gamma _m}}}/{{[j( {\omega - {\omega _m}} ) + {\Gamma _m}]\rbrace }}} }} \\
&=& \frac{1}{{\sum _m {{(1}/{{{Z_m})}}} }},
\end{eqnarray*}

where


(7) \begin{eqnarray*}
{Z_m} = \frac{{j( {\omega - {\omega _m}} ) + {\Gamma _m}}}{{{\gamma _m}}}
\end{eqnarray*}

denotes the impedance of the *m*th mode. The above equations reveal the interdependence between the characteristics of the modes (resonant frequency, intrinsic loss and radiation loss) and the response of the system (reflection spectrum or absorption spectrum), where the absorption coefficient is given by


(8) \begin{eqnarray*}
\alpha = 1 - {| r |^2}.
\end{eqnarray*}

These features and system responses are mutually determined by each other. (The inverse deduction of resonance parameters from the absorption spectrum is detailed in Note S3C). In Fig. 1(b), the blue curves represent the absorption spectra achieved by employing 80 modes ($N=80$) and finely modulating the resonance parameters of each mode. This modulation aims to achieve a strong *Q*-weighted mode density and ultrabroadband quasi-perfect absorption. Conversely, the purple curves correspond to absorption spectra obtained when the parameters deviate from the modulation. Furthermore, due to the considerable number of parameters involved, optimization algorithms like genetic algorithms are employed to facilitate the design process. Figure 1(c) is obtained by substituting the parameters of the modes into (1). By fixing the resonance frequency (i.e. maintaining an unchanged mode density), and varying the radiative and intrinsic losses to $\zeta$ times their original values [as indicated by (1), which demonstrates a positive correlation with $\zeta$], we can observe the impact of the extent of deviation in resonance parameters on both the absorption spectrum and the coupling strength (see Fig. S1), with the coupling strength defined in [15].

### Impedance calculation of the wire mesh

The wire mesh is a nearly zero-thickness (relative to the manipulated wavelength) resistive material whose impedance ($z = r + jx$) can be expressed as [54,55]


(9) \begin{eqnarray*}
{r_a} = \frac{{1.166}}{{100}}\frac{{{{( {1 - \sqrt{{\sigma _e}} } )}^2}}}{{{d_w}}},
\end{eqnarray*}

(10) \begin{eqnarray*}
{x_a} = \frac{{2{d_w} + 0.52{s_w}\sqrt{{\sigma _e}} }}{{1000{\sigma _e}}}k,
\end{eqnarray*}

with an effective open area ratio of mesh


(11) \begin{eqnarray*}
{\sigma _e} &=& \bigg ( {1 - \frac{{{d_w}}}{{{s_w}}}} \bigg )\bigg [ {\sqrt{1 + {{\bigg ( {\frac{{{d_w}}}{{{s_w}}}} \bigg )}^2}} - \frac{{{d_w}}}{{{s_w}}}} \bigg ] \\
&& \times ( {0.95 + 3.656{d_w}} ),
\end{eqnarray*}

where $r_a$ and $x_a$ represent the acoustic resistance and reactance, respectively, $d_w$ is the wire diameter, $s_w$ is the wire spacing of the wire mesh (see Fig. S10) and *k* denotes the wave number. The mesh count of the wire mesh ${C_m} = {{1\,\,{\mathrm{inch}}} / {{s_w}}}$. The thickness of the wire mesh ${t_w} = 2{d_w}$. A functional relationship between $d_w$ and $C_m$ can be fitted by measuring $t_w$ for different $C_m$ (see Fig. S10). The impedance of the wire mesh as a function of $C_m$ can be obtained by substituting the $d_w$ and $s_w$ relationships with $C_m$ into (9)–(11). The parameters corresponding to $C_m = 500$ are $d_w = 0.025$ mm, $t_w = 0.05$ mm and $s_w = 0.051$ mm, and according to (9)–(11), ${\sigma _{\rm {e}}} = 0.331$, ${r_a} = 8.41 \times {10^{ - 2}}$ and $x_a / k = 1.97 \times {10^{ - 4}}$. Moreover, the critical frequency for (9)–(11) failure is more than 20 000 Hz, which is much larger than $f_{\rm {max}}$ [54]. In addition, the incident sound pressure level in the experiment is much lower than 120 dB, so the nonlinear effect of the wire mesh does not need to be considered. The near-zero thickness and negligible acoustic reactance properties of the wire mesh permit decoupling and regulation of the radiation and intrinsic losses of resonators (see Note S4 for details), a capability that is not readily achievable with other materials in acoustics. Additionally, in practical flow applications (such as aeroengine noise reduction), the wire mesh effectively suppresses secondary noise generation by reducing turbulent energy and dissipating flow kinetic energy, thereby preventing degradation of the absorber’s noise reduction performance. In the field of electromagnetic waves, graphene layers are promising as analogous resistive materials [40]. Integrating the strategy proposed in this work could enable broader-band and more efficient wave absorption.

### Experimental setup

The experimental platform shown in Fig. 4(a) consists of a sound source, power amplifier, data acquisition card (DAC), computer and improved SWT. The sound source is a BMS 5599 loudspeaker. The power amplifier amplifies the white noise signal set by the program and transmits it to the sound source for sound generation. The DAC has two NI PXIe-4497 acquisition cards with 16 channels, which are capable of simultaneously extracting up to 32 channels of sound field data and returning it to the program for pre-processing. The improved SWT consists of square-section standing-wave tubes T102 and T50 with side lengths of 102 and 50 mm, respectively, and an adapter tube Ta. Tube T102 is matched to the size of the source to be received, and Ta connects T102 to T50 and directs the sound to T50. Tube T50 has a plane-wave-only cutoff frequency (the highest frequency that can be measured by the previous SWT) of 3432 Hz. Its measurement section houses 32 1/4-inch GRAS-type 46BD microphones (see Fig. S21), the placements of which are reported in Table S4. Since the target frequency band is too wide, considering the limitation of the sound source, we divided it into four frequency bands of 80–400 Hz, 400–3432 Hz, 3432–8000 Hz and 8000–12 800 Hz measurements. After the sound source has reached a stable state, the 32 microphone data are transmitted through the 32 channels of the DAC acquisition. A total of 50 measurements are taken for each group of data, and the average is calculated. The sound field information extracted into the program is pre-processed for the measurement of the frequency of each point of the amplitude of sound pressure and the phase of the information; the subsequent processing and analysis of the data are described in detail in Note S9.

In both theoretical and simulation analyses, the walls of the sample are treated as acoustic hard boundaries. For the experimental setup, the samples were fabricated using 3D-printed resin, with a density of $\rho _m=1300$ $\rm {kg/m^3}$ and a sound speed of $c_m=2700$ $\rm {m/s}$. In comparison, the density and sound speed of air are $\rho _0=1.21$ $\rm {kg/m^3}$ and $c_0=343$ $\rm {m/s}$, respectively. The characteristic impedance ratio between the resin and air is calculated as $R_I=\rho _mc_m/\rho _0c_0\approx 8457$, which is much greater than 1. This significant impedance contrast demonstrates that the walls of the experimental samples can be effectively considered as acoustic hard boundaries.

## Supplementary Material

nwaf199_Supplemental_File
