# Supplementary material for: Seven-octave ultrabroadband metamaterial absorbers via quality-factor-weighted mode density modulation
Source: Natl Sci Rev. 2025 May 20;12(7):nwaf199. doi: 10.1093/nsr/nwaf199 (PMC12218206; doi:10.1093/nsr/nwaf199)
Supplement: nwaf199_Supplemental_File [file nwaf199_supplemental_file.pdf]

Supplementary Materials for

Seven-octave ultrabroadband metamaterial absorbers via Q-weighted mode density modulation

Nengyin Wang[1,†], Sibo Huang[2,†], Zhiling Zhou[1,†],

Din Ping Tsai[2]*, Jie Zhu[1]*, Yong Li[1]*

[1]Institute of Acoustics, Tongji University, Shanghai 200092, China

[2]Department of Electrical Engineering, City University of Hong Kong, Hong Kong 999077, China

*dptsai@cityu.edu.hk *jiezhu@tongji.edu.cn *yongli@tongji.edu.cn

[†]These authors contributed equally to this work.

Contents

Note 1: Analyzing the influence of XQMD on absorption and coupling.

Note 2: Details related to the approaches for modulating absorption.

Note 3: Details about absorber design theory.

Note 4: Detailed theory and comparison related to wire mesh.

Note 5: Oblique incidence absorption performance of MMA.

Note 6: Details related to the minimum thickness of the causal constraints.

Note 7: Comparison of MMA and conventional absorber performance.

Note 8: Comparison of different MMAs.

Note 9: Details related to experimental theory.

Note 1: Analyzing the influence of XQMD on absorption and coupling.

The MRM2 system comprises 80 resonant modes with resonance frequency vector $\boldsymbol{\omega}2$, radiative loss vector $\boldsymbol{\gamma}2$, and intrinsic loss vector $\boldsymbol{\Gamma}2$. Taking this configuration as a benchmark, the resonant frequency is fixed and varying the radiative and intrinsic losses to ζ times of the original values, i.e. $\boldsymbol{\gamma} = \zeta\boldsymbol{\gamma}2$ and $\boldsymbol{\Gamma} = \zeta\boldsymbol{\Gamma}2$. According to eq. (1), χ_{QMD} is positively correlated with ζ, where $\zeta = 1/5$ corresponds to the MRM1 system. As ζ increases, the absorption performance of the MRM system improves and the coupling strength intensifies (Fig. S1).

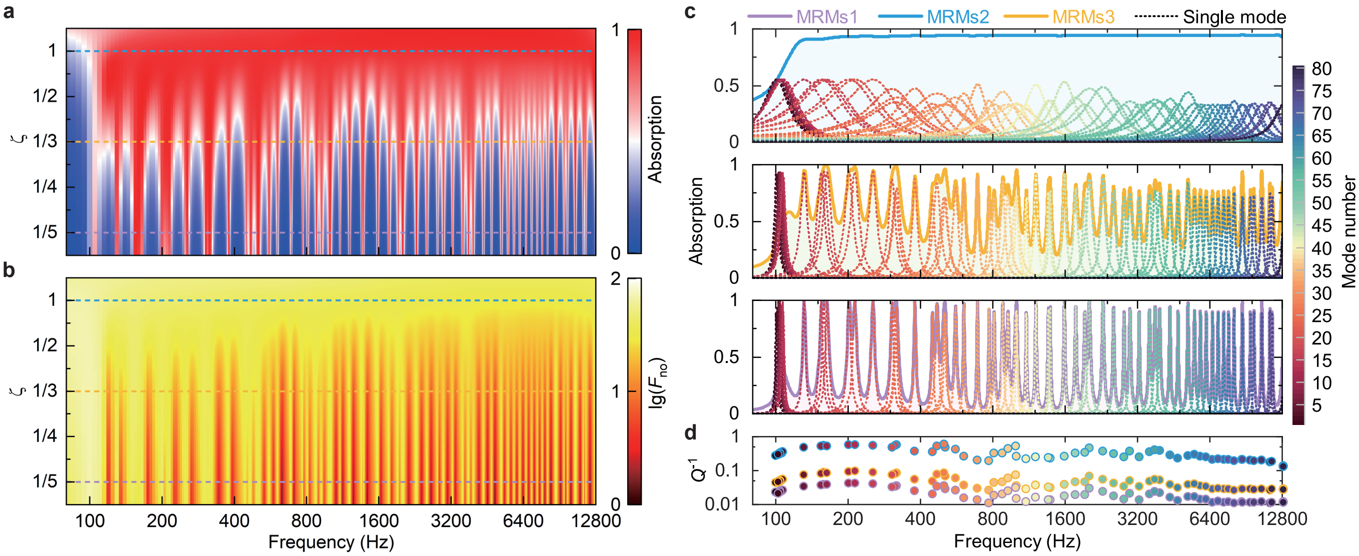

Fig. S1 | Influence of χ_{QMD} on absorption strength and magnitude of nonlocal effects in coupled systems of MRMs. **a**, As ζ increases, the absorption performance of the MRMs system improves. **b**, As ζ increases, the nonlocal effect also increases, meaning that the coupling between modes becomes stronger. **c**, The overall absorption spectra of MRMs corresponding to $\zeta = 1$ (top), $\zeta = 1/3$ (middle), and $\zeta = 1/5$ (bottom) (solid lines) and absorption spectra of individual modes (dashed lines). As ζ increases, the modes in the system show a transition from strong absorption and weak coupling (strong oscillations in the overall absorption spectra) to weak absorption and strong coupling (excellent overall absorption performance and smooth spectra). The individual modes are finely modulated for $\zeta = 1$. $\zeta = 1/3$ and $\zeta = 1/5$ are the same parameters as Fig. 1b and 1c in the main text. **d**, The distribution of Q^{-1} for the modes corresponding to the three systems.

Note 2: Details related to the approaches for modulating absorption.

A. Resonance modulation via embedded necks and wire mesh.

The wire mesh predominantly modulates the intrinsic loss with minimal impact on radiative loss, thereby driving more resonant modes into the targeted over-damped regime and enhancing Q^{-1} (Fig. S2). Combined with the embedded neck structure, both intrinsic and radiative losses can be independently tuned across a broader spectrum.

The embedded-neck intermediate layer extends the acoustic wave propagation path in MCNEHR, effectively increasing the longitudinal acoustic path length. Given the inverse correlation between resonance frequency and the resonator's equivalent length, this configuration shifts resonant modes toward lower frequencies while generating additional higher-order resonant modes (Fig. S3).

B. Geometric parameters and simulation of the structure in Fig. 2.

A comparison between the absorption coefficients and acoustic impedances obtained from COMSOL simulations and theoretical analyses shows excellent agreement (Fig. S4), confirming the accuracy of the proposed theory. The detailed geometric parameters of the structure are provided in Tables S1 and S2.

Sample No.	Layer No.	l_u	d	l_c	l_d
s1	1	15	10	78	0
s2	1	15	10	38	0
	2	15	8	40	10
s3	1	15	10	25	0
	2	15	8	26	5
	3	15	4	27	5

Table S1. The geometry parameters (in mm) of the MCNEHR samples of single-layer (s1), double-layer (s2) , and three-layer (s3) in Fig2A. The length and width of the samples are 24 mm.The definition of the parameters is shown in Fig. S6.

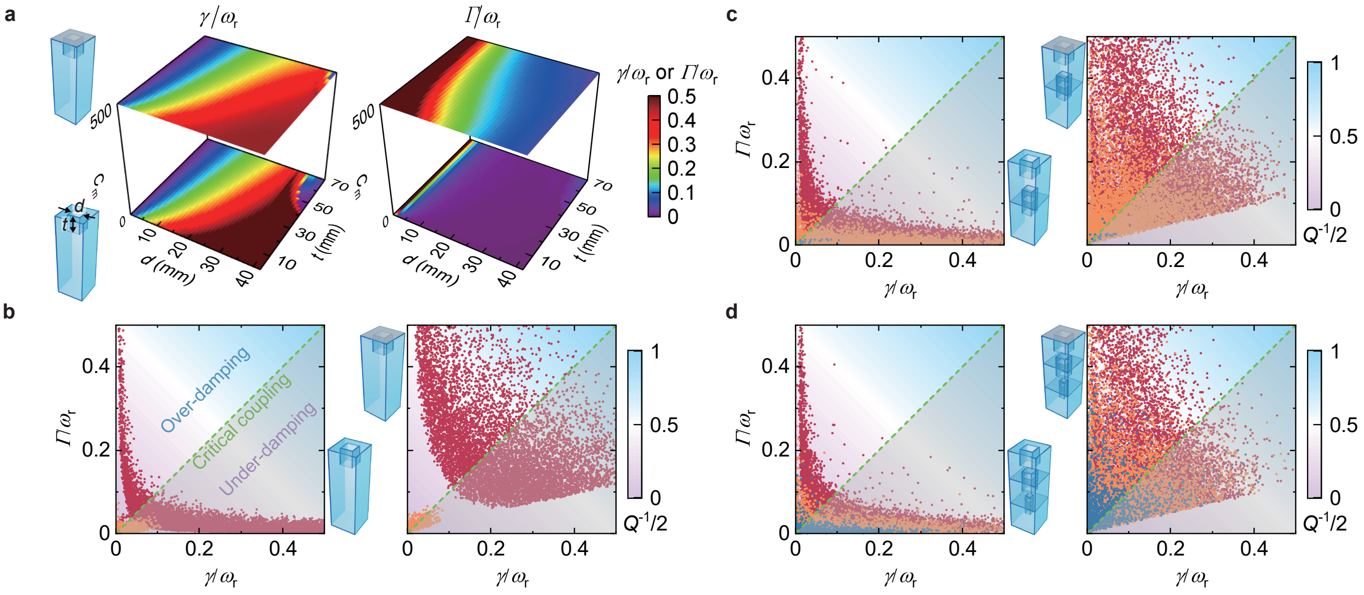

Fig. S2 | Modulation of intrinsic and radiative losses by embedded necks and wire mesh. a, The radiative loss (left panel) and intrinsic loss (right panel) of MCNEHR with $C_m = 0$ and $C_m = 500$ varying lengths d and heights t of the embedded neck. **b-d**, The phase diagram of intrinsic and radiative losses for one layer (**b**), two layers (**c**), three layers (**d**) of MCNEHR. The color scale represents the sum of the horizontal and vertical coordinates, which corresponds to $Q_n/2 = \Gamma_n/\omega_n + \gamma_n/\omega_n$ positively correlated with χ_{QMD}. The red/orange/blue dots indicate 1st/2nd/3rd order resonances, respectively. The MCNEHR without wire mesh covering (left panel, $C_m = 0$) can only achieve intrinsic and radiative losses in a narrow range, while the MCNEHR with wire mesh covering (right panel, $C_m = 500$) is capable of modulating intrinsic and radiative losses in a broad range.

Unit No.	Layer No.	l_u	d	s	l_c	h	l_d
1	1	9.5	10.1	47.7	78	17	0
2	1	16	11.3	47.7	38	29.7	0

Table S2. The geometry parameters (in mm) of the samples of the two units in Fig. 2C. Here, s and h represent the length and width of the unit cavity, respectively, while the definitions of the remaining parameters are shown in Fig. S6.

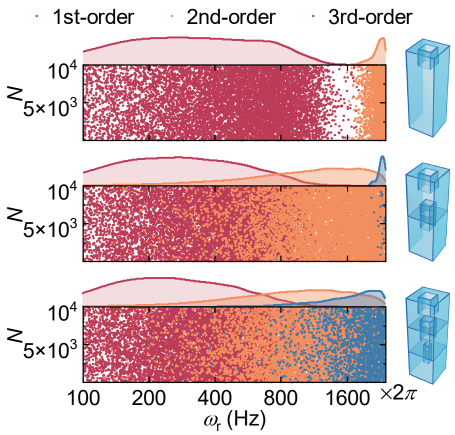

Fig. S3 | Distribution of resonance frequencies for different layers of MCNEHR. The three panels from top to bottom show the distribution of resonance frequencies corresponding to one layer, two layers, and three layers of MCNEHR. The red/orange/blue dots indicate 1st/2nd/3rd order resonances, respectively. The data for each panel are obtained from 10^4 randomly generated sets of MCNEHR that satisfy the geometric constraints.

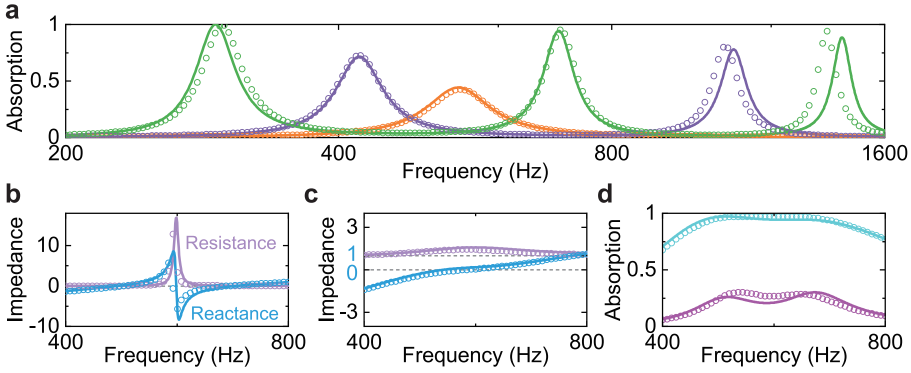

Fig. S4 | Comparison of theoretical and simulated data for absorption spectra and impedance spectra in Fig. 2. **a**, Absorption spectra of single-, double-, and three-layer HR structures: theoretical (solid lines) vs. simulated (hollow circles). **b**, Acoustic resistance (purple) and reactance (blue) of the dual-cavity structure without metal wire mesh: theoretical (solid lines) vs. simulated (hollow circles). **c**, Acoustic resistance (purple) and reactance (blue) of the dual-cavity structure with 500-mesh metal wire mesh: theoretical (solid lines) vs. simulated (hollow circles). **d**, Absorption spectra of the dual-cavity structure without (magenta) and with (cyan) 500-mesh metal wire mesh: theoretical (solid lines) vs. simulated (hollow circles).

Note 3: Details about absorber design theory.

A. Mode matching method for calculating absorption.

Since the actual structure must satisfy geometrical constraints and spatial arrangement, specific methods are required for different wave systems to design the corresponding MMAs. The mode matching method (this section) and the transfer matrix method (next section) are employed to calculate the acoustic response (e.g., absorption, reflection) of the acoustic MMAs demonstrated in the main text.

The manipulation of mixed modes is crucial, as the absorption characteristics of absorbers in practical applications tend to be closer to their performance under mixed-mode incidence. However, due to its complexity, previous research has largely sidestepped the investigation of mixed-mode absorption properties. To efficiently and accurately control the performance of MMAs across seven octaves of frequency bands, it is imperative to consider their absorption properties under mixed-mode incidence, transcending the limitations of traditional impedance calculation models and finite element simulations. Impedance calculation models require the absorber's cross-section to be within the subwavelength range, where the lateral dimension of the absorber is proportional to the smallest wavelength, and the longitudinal length of the absorber is proportional to the largest wavelength for broadband absorption. When the maximum and minimum wavelengths of the target frequency band differ by more than two orders of magnitude, the cross-sectional size of the absorber becomes significantly smaller than the longitudinal dimension of sound wave propagation, which is detrimental to both the construction and practical usage of the absorber. While finite element simulation methods can offer more detailed numerical solutions, their computational efficiency and resource consumption pose non-negligible bottlenecks. Specifically, when dealing with wide frequency bands and large wavelength differences, the demand for mesh refinement soars, leading to an exponential increase in computational load. This imposes stringent requirements on computing resources and hinders the effective optimization of multi-degree-of-freedom sound-absorbing structures.

Therefore, we have developed an efficient, accurate and fast theoretical calculation method based on the mode matching method and the transfer matrix method that can compute the complex case containing mixed modes. The mode matching method can accurately compute the sound field for multiple wave modes propagation, while the transfer

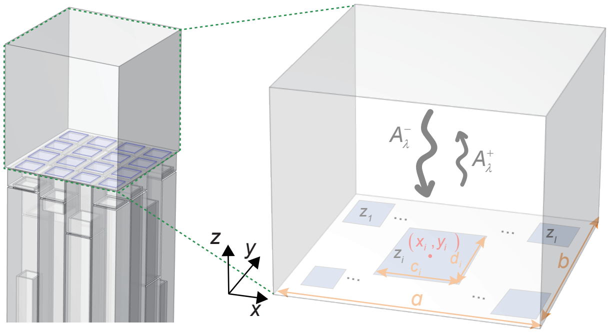

Fig. S5 | Schematic of the model matching method. The left panel is a perspective view including the actual structure, and the right panel is the simplified model schematic, where the blue region denotes the impedance z_i at the aperture of the subunit (MCNEHR in this paper) of the absorber, the incident acoustic wave in the external region is A_λ^- and the reflected acoustic wave is A_λ^+, and the subscript λ denotes the order of the wave mode. (x_i, y_i), c_i and d_i denote the center position, length and width of the i-th aperture, respectively. a and b denote the length and width of the external region (the length and width of the hard boundary, or the length of the period of the periodic boundary in the x and y directions).

matrix method can efficiently improve the computation time. The modulation of the MMA to the external acoustic field (including incident and reflected waves) can be solved by the mode matching method (Fig. S5). According to the Helmholtz equation, the external acoustic field can be expressed as the superposition of different waveguide modes, and the acoustic pressure can be written as

$$p = \sum_{\lambda_{m,n}}^{\infty} \mathrm{X}_{\lambda_{m,n}}(x, y)(A_{m,n}^- e^{jk_{z,\lambda_{m,n}} z} + A_{m,n}^+ e^{-jk_{z,\lambda_{m,n}} z}), \tag{S1}$$

and the corresponding particle velocity field in the z-direction can be written as

$$v_z = \frac{1}{\rho_0 \omega} \sum_{\lambda_{m,n}}^{\infty} \mathrm{X}_{\lambda_{m,n}}(x, y) k_{z,\lambda_{m,n}} (A_{m,n}^+ e^{-jk_{z,\lambda_{m,n}} z} - A_{m,n}^- e^{jk_{z,\lambda_{m,n}} z}). \tag{S2}$$

Where ρ_0 is the density of the medium in the external region (above the absorber), and ω is the angular frequency. $A_{m,n}^{+(-)}$ corresponds to the (m, n)-th order incident (reflected) wave amplitude. $\mathrm{X}_{\lambda_{m,n}}$ is the system of eigenfunctions determined by the boundary conditions in the x-axis and y-axis directions. The hard boundary corresponds to $\mathrm{X}_{\lambda_{m,n}}(x, y) = \cos(k_{x,m} x) \cos(k_{y,n} y)$, and the periodic boundary corresponds to $\mathrm{X}_{\lambda_{m,n}}(x, y) =$

$e^{-jk_{y,n}y}e^{-jk_{x,m}x}$, where $k_{x,m}$, $k_{y,n}$, and $k_{z,\lambda_{m,n}}$ correspond to the x, y, and z components of the wavevector, respectively, in the detailed form that can be found in our previous work ref. 45. In this work, the analytical calculations and experiments correspond to hard boundary conditions. For calculations of oblique incidence characteristics, periodic boundary conditions were used (see Fig. S16).

At the absorber surface according to the continuity of acoustic pressure and particle velocity can be obtained

$$p^{\mathrm{I}} = p_i^{\mathrm{II}},\ (x,y) \in \Omega_i \tag{S3}$$

and

$$v_z^{\mathrm{I}} = \begin{cases} v_{z,i}^{\mathrm{II}},\ (x,y) \in \Omega_i \\ 0, \text{other region} \end{cases} \tag{S4}$$

where $\Omega_i = \{(x,y)\,|x_i - c_i/2 < x < x_i + c_i/2, y_i - d_i/2 < y < y_i + d_i/2\}, i = 1, 2, \cdots, I$ denotes the aperture impedance region, (x_i, y_i), c_i and d_i denote the center position, length and width of the i-th aperture, respectively (see Fig. S5). The other regions are hard boundary regions, i.e., impedance infinity. For the sake of illustration, we denote the external region as region I, and the region of the absorber's aperture as region II. The impedance z_i at the aperture of the i-th MCNEHR unit of the MMA can be calculated using the transfer matrix method (see next section). From the definition of impedance and eqs. S3 and S4, we have

$$v_{z,i}^{\mathrm{II}}(x,y) = \frac{p_i^{\mathrm{II}}(x,y)}{z_s^i(x,y)} = \frac{p^{\mathrm{I}}(x,y)}{z_s^i(x,y)} = \sum_{\lambda_{m,n}}^{\infty} \frac{\mathrm{X}_{\lambda_{m,n}}(x,y)}{z_s^i(x,y)}(A_{m,n}^{-}e^{jk_{z,\lambda_{m,n}}z} + A_{m,n}^{+}e^{-jk_{z,\lambda_{m,n}}z}), \tag{S5}$$

substituted into eq. S5, and using the orthogonal complete eigenfunction set $\mathrm{X}_{\lambda_{m,n}}$, weighted integrals can be obtained as

$$N_1\left(\vec{A_\lambda^+} - \vec{A_\lambda^-}\right) = N_2 II\left(\vec{A_\lambda^+} + \vec{A_\lambda^-}\right), \tag{S6}$$

with

$$\begin{aligned} &N_1(\lambda_{m'n'}, \lambda_{m,n}) = \tfrac{k_{z,\lambda_{m,n}}}{ab}\int_0^a\int_0^b \mathrm{X}_{\lambda_{m,n}}(x,y)X_{\lambda_{m',n'}}(-x,-y)\,dxdy, \\ &N_2^i(\lambda_{m'n'}, \mu_{k,l}) = \tfrac{\rho_0\omega}{ab}\int_{x_i-\frac{c_i}{2}}^{x_i+\frac{c_i}{2}}\int_{y_i+\frac{d_i}{2}}^{y_i+\frac{d_i}{2}} \tfrac{\mathrm{X}_{\lambda_{m,n}}(x,y)}{z_s^i(x,y)}X_{\lambda_{m',n'}}(-x,-y)dxdy, \\ &N_2(\vec{\lambda_{m'n'}}) = \begin{pmatrix} N_2^1(\vec{\lambda_{m'n'}}) & N_2^2(\vec{\lambda_{m'n'}}) & \cdots & N_2^i(\vec{\lambda_{m'n'}}) & \cdots & N_2^I(\vec{\lambda_{m'n'}}) \end{pmatrix}, \end{aligned} \tag{S7}$$

and

$$\begin{aligned} &\vec{A_\lambda^\pm} = \begin{pmatrix} A_{\lambda_{-M,-N}}^\pm & A_{\lambda_{-M+1,-N}}^\pm & \cdots & A_{\lambda_{m,n}}^\pm & \cdots & A_{\lambda_{M,N}}^\pm \end{pmatrix}^T, \\ &II = \begin{pmatrix} I_{NM} & I_{NM} & \cdots & I_{NM} & \cdots & I_{NM} \end{pmatrix}^T, \end{aligned} \tag{S8}$$

where I_{NM} is the unit matrix. a and b denote the length and width of the region I. Equation 17 can be rewritten as a system of non-homogeneous linear equations

$$(N_1 - N_2 II) \overrightarrow{A_\lambda^+} = (N_2 II + N_1) \overrightarrow{A_\lambda^-}, \tag{S9}$$

where the coefficient matrices N_1 and N_2 are determined by structural parameters and boundary conditions, and the reflected wave amplitude $\overrightarrow{A_\lambda^+}$ can be solved by eq. S9 for a given incident sound pressure $\overrightarrow{A_\lambda^-}$.

Since there are mixed propagation modes in the outer region, the absorption coefficient cannot be calculated simply from the reflection coefficient of the plane wave, here we directly calculate the absorption of the MMA by using the reflected energy W_{re} and the incident energy W_{in}, i.e., the absorption coefficient is defined as

$$\alpha = 1 - \left| \frac{W_{\mathrm{z,re}}}{W_{\mathrm{z,in}}} \right|. \tag{S10}$$

Where the energy

$$W_{\mathrm{z}} = \frac{1}{ab} \int_0^b \int_0^a I_{\mathrm{z}} dx dy, \tag{S11}$$

with z-direction energy flow $I_z = 1/2 * \mathrm{Re}(p\bar{v}_{\mathrm{z}})$. Substituting eqs. S1 and S2 to eq. S11 and simplify to obtain

$$W_{\mathrm{z,in(re)}} = \frac{\delta \left| \overrightarrow{A^{-(+)}} \right|^2}{2\rho_0 \omega} * \mathrm{Re}\left(\overrightarrow{k_{\mathrm{z}}} \right), \tag{S12}$$

where for hard boundary $\delta \left|\overrightarrow{A^-}\right|^2 (\lambda_{m,n}) = ((1 + \delta_{n,1})/2) ((1 + \delta_{m,1})/2) \left| A_{m,n}^- \right|^2$ and for periodic boundary $\delta \left|\overrightarrow{A^-}\right|^2 (\lambda_{m,n}) = \left| A_{m,n}^- \right|^2$.

B. Transfer matrix method for calculating MCENHR impedance.

The MCNEHR consists of a cascade of multilayer similar structures (see Fig. S6). The single-layer similar structure (as shown by the red dashed box in Fig. S6) contains the embedded neck (EN) and cavity. Therefore, when using the transfer matrix method to calculate the impedance at the aperture of the MCNEHR, it is only necessary to derive the transfer matrix of the single-layer structure, while substituting the corresponding structural parameters for the different layers. The transfer matrix at different positions within the single-layer structure can be derived from the continuity of the sound pressure (P) and the volume velocity (V). Several equations involved can be expressed as

$$\begin{pmatrix} P_{z_h} \\ V_{z_h} \end{pmatrix} = M_{z_h} \begin{pmatrix} P_{z_{h-1}} \\ V_{z_{h-1}} \end{pmatrix}, \tag{S13}$$

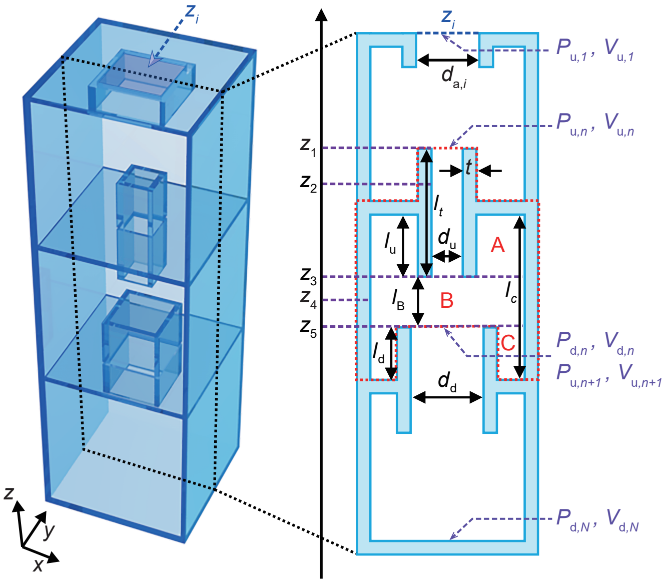

Fig. S6 | Schematic of the MCNEHR. The left panel shows the 3-D perspective schematic of MCNEHR. The right panel shows the longitudinal section of MCNEHR (black dashed box in the left panel) to demonstrate the transfer matrix method for solving the impedance at the aperture of MCNEHR. MCNEHR can be viewed as a cascade of multilayers with similar structures consisting of embedded neck and cavity (shown in the red dashed box). The cavity can be divided into three regions, A, B, and C.

when h takes different numbers (2, 3, 4, 5), M_{z_h}, $P_{z_h(z_{h-1})}$, and $V_{z_h(z_{h-1})}$ denote the transfer matrix, sound pressure and volume velocity at z_1, z_2, z_3, z_4, z_5, respectively. The specific transfer matrix is given below. At the upper port of EN (z_1), the end correction of the acoustic impedance can be added by employing the transfer matrix

$$M_{\mathrm{EN_{up}}} = \begin{bmatrix} 1 & jk\left(\delta - j\sqrt{2\eta/\omega\rho_0}\right) Z_0/S_{\mathrm{EN}} \\ 0 & 1 \end{bmatrix}, \tag{S14}$$

where k, ω, ρ_0, and Z_0 are the wave number, angular frequency, mass density, and characteristic impendence of air in free space, respectively. η is the dynamic viscosity of static air. δ is the end correction of EN relative to the cavity. $S_{\mathrm{EN}} = {d_u}^2$ is the area of the square section of EN in the xy-plane. Inside the channel of EN (z_2), the transfer matrix is

$$M_{\mathrm{EN}} = \begin{bmatrix} \cos(k_{\mathrm{EN}} l_t) & j\sin(k_{\mathrm{EN}} l_t)\, Z_{\mathrm{EN}}/S_{\mathrm{EN}} \\ j\sin(k_{\mathrm{EN}} l_t)\, S_{\mathrm{EN}}/Z_{\mathrm{EN}} & \cos(k_{\mathrm{EN}} l_t) \end{bmatrix}, \tag{S15}$$

where k_{EN} and $Z_{\mathrm{EN}} = \rho_{\mathrm{EN}} c_{\mathrm{EN}}$ are the complex wave number and complex characteristic

impedance of air inside EN. For the lower port of EN the same has

$$M_{\mathrm{EN_{down}}}=\begin{bmatrix} 1 & jk\left(\delta - j\sqrt{2\eta/\omega\rho_0}\right)Z_0/S_{\mathrm{EN}} \\ 0 & 1 \end{bmatrix}, \tag{S16}$$

Secondly, the wave from EN to the cavity is split into two flow directions to the two regions A and B at z_3. The corresponding transfer matrix is

$$M_{\mathrm{AB}}=\begin{bmatrix} 1 & 0 \\ j\tan\left(k_{\mathrm{A}}l_{\mathrm{u}}\right)S_{\mathrm{A}}/Z_{\mathrm{A}} & 1 \end{bmatrix}, \tag{S17}$$

where k_{A} and $Z_{\mathrm{A}} = \rho_{\mathrm{A}}c_{\mathrm{A}}$ are the complex wave number and complex characteristic impedance of region A, and $S_{\mathrm{A}} = S_{\mathrm{B}} - (d_u + t)^2$ is the cross-sectional area of region A, S_{B} is the cross-sectional area of region B. Similarly, the transfer matrix at inside region B (z_4) and the upper port of the next layer EN (z_5) can be obtained as

$$M_{\mathrm{B}}=\begin{bmatrix} \cos\left(k_{\mathrm{B}}l_{\mathrm{B}}\right) & j\sin\left(k_{\mathrm{B}}l_{\mathrm{B}}\right)Z_{\mathrm{B}}/S_{\mathrm{B}} \\ j\sin\left(k_{\mathrm{B}}l_{\mathrm{B}}\right)S_{\mathrm{B}}/Z_{\mathrm{B}} & \cos\left(k_{\mathrm{B}}l_{\mathrm{B}}\right) \end{bmatrix}, \tag{S18}$$

and

$$M_{\mathrm{BC}}=\begin{bmatrix} 1 & 0 \\ j\tan\left(k_{\mathrm{C}}l_{\mathrm{d}}\right)S_{\mathrm{C}}/Z_{\mathrm{C}} & 1 \end{bmatrix}, \tag{S19}$$

where $k_{\mathrm{B(C)}}$ and $Z_{\mathrm{B(C)}} = \rho_{\mathrm{B(C)}}c_{\mathrm{B(C)}}$ are the complex wave number and complex characteristic impedance of region B (C). $S_{\mathrm{C}} = S_{\mathrm{B}} - (d_d + t)^2$ is the cross-sectional areas of region C.

Then the total transfer matrix for the n-th layer T_n can be expressed as

$$T_n = M_{\mathrm{EN_{up}},n}M_{\mathrm{EN},n}M_{\mathrm{EN_{down}},n}M_{\mathrm{AB},n}M_{\mathrm{B},n}M_{\mathrm{BC},n}, \tag{S20}$$

which leads to

$$\begin{pmatrix} P_{\mathrm{u},n} \\ V_{\mathrm{u},n} \end{pmatrix} = T_n \begin{pmatrix} P_{\mathrm{d},n} \\ V_{\mathrm{d},n} \end{pmatrix}. \tag{S21}$$

Nevertheless, it should be noted that the utilization of the mode matching method in calculating the overall structure and the external sound field region inherently accounts for the higher modes. As a result, there exists no necessity for an end correction at the upper port of the EN of the first layer, i.e.,

$$M_{\mathrm{EN_{up}},1}=\begin{bmatrix} 1 & k\sqrt{2\eta/\omega\rho_0}Z_0/S_{\mathrm{EN}} \\ 0 & 1 \end{bmatrix}. \tag{S22}$$

Finally, the transfer matrix for the entire MCNEHR is

$$T = \sum_{n=1}^{N} T_n = \begin{bmatrix} T_1 & T_2 \\ T_3 & T_4 \end{bmatrix}. \tag{S23}$$

Combined with the hard boundary condition at the bottom of the last layer, i.e., $V_{\mathrm{d},N} = 0$, the acoustic impedance of the MCNEHR necessary for the previous section can be denoted as $z_i = T_1/T_3(d_{\mathrm{a},i})^2$.

When a wire mesh is covered to the surface of the MMA, the negligible thickness of the mesh allows for a simple superposition of its impedance (z_{wm}) with that of the MCHEHR (z_{MCNEHR}), resulting in the impedance of a single unit represented as $z = z_{\mathrm{wm}} + z_{\mathrm{MCNEHR}}$.

C. Extraction of resonance parameters of the modes via absorption spectrum of the unit.

From Methods section 'CMT for MRM', when N = 1

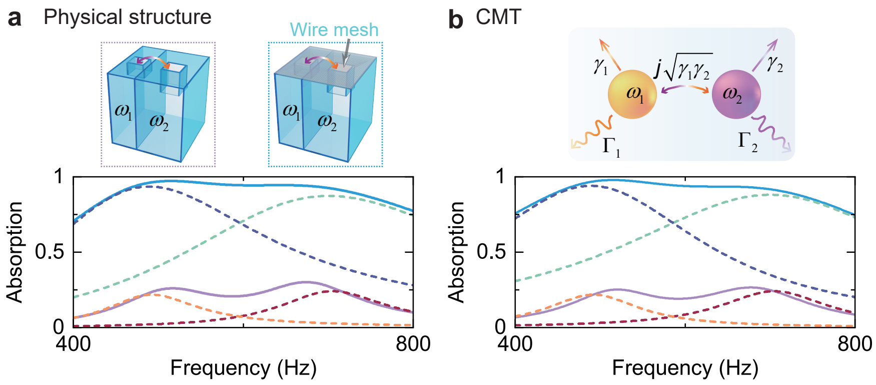

Fig. S7 | Comparison between acoustic theoretical models and CMT predictions for double-cavity absorption performance in Fig.2c. a, Physical structure performance: Absorption spectra for single-cavity (dashed) and coupled double-cavity (solid) configurations without (purple, orange, red) and with (blue, dark blue, green) wire mesh, calculated via the mode-matching method (Supplementary Note 3A) and impedance transfer theory (Supplementary Note 3B). **b**, CMT predictions: Absorption spectra derived by feeding single-cavity resonance parameters (ω, Γ, γ) extracted via parameter extraction (Supplementary Note 3C) into the "CMT for MRM" framework, for single-cavity (dashed) and coupled double-cavity (solid) cases without (purple, orange, red) and with (blue, dark blue, green) wire mesh. The agreement between theoretical methods validates both the CMT model and the coupling term $\sqrt{\gamma_n \gamma_m}$.

$$\alpha = \frac{4\Gamma_1\gamma_1}{(\Gamma_1+\gamma_1)^2+(\omega-\omega_1)^2}. \tag{S24}$$

Thus the absorption peak is at the resonant frequency $\omega = \omega_1$. At this point, the impedance is

$$Z_1 = r_z = \frac{\Gamma_1}{\gamma_1}. \tag{S25}$$

The corresponding half-high bandwidth

$$\Delta\omega = 2\left(\Gamma_1+\gamma_1\right). \tag{S26}$$

Consequently, by leveraging the acoustic resistance at resonance and the aforementioned half-height bandwidth, one can accurately ascertain the radiation and intrinsic loss of the mode, denoted as

$$\begin{cases} \gamma_1 = \frac{\Delta\omega}{2(r_z+1)} \\ \Gamma_1 = \frac{r_z\Delta\omega}{2(r_z+1)} \end{cases}. \tag{S27}$$

The distribution of Q^{-1} and χ_{QMD} in Fig. 2 and Fig. 3 can be obtained by calculating the absorption spectra of the subunits of the MMA.

Taking the double-cavity structure in Fig. 2C as an example, the absorption spectrum predicted by CMT using the extracted resonator parameters shows good agreement with the theoretical calculation results presented above (Fig. S7).

D. The workflow for designing absorbers based on the theory presented in this work.

Step 1 (universal for all wave systems): Define the desired absorption spectrum and determine the level of structural complexity (e.g., number of resonators) and use coupled mode theory (see Methods section 'CMT for MRM') to calculate the required mode density and the Q-weighted mode density distribution.

Step 2: Implement the desired mode distribution within the specific wave system, including the following steps (for acoustic systems):

1) Establish the impedance model bridging the structural parameters of each unit and its frequency response (see Supplementary Materials Note 3B).

2) Use the resonance parameter extraction method (see Supplementary Materials Note 3C) to link structural impedance to essential qualities of resonant mode (resonance frequency, intrinsic loss, and radiative loss).

3) Optimize the structural parameters to reach the desired impedance and mode properties.

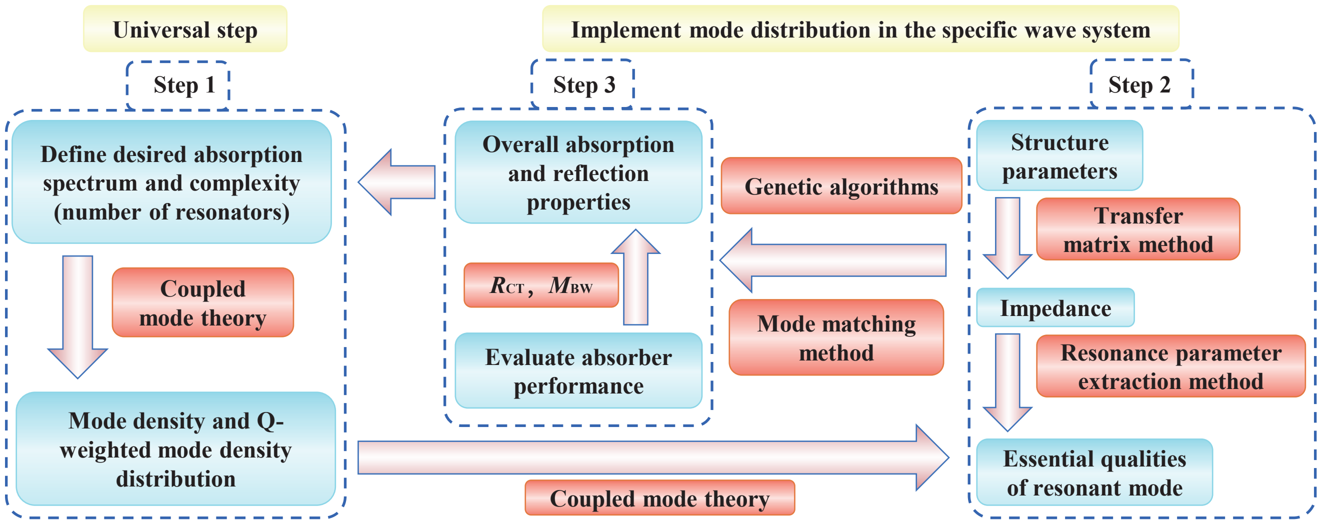

Fig. S8 | Flowchart for absorber design workflow. The flowchart consists of universal step (applicable to all fluctuating systems) and the implementation of the methodology specifically in wave systems (in the case of the acoustic absorber design, other wave systems just need to replace the various parts of the design flow with the corresponding methodology), where the concepts and theories presented in the article are tied together. See note 3D for a detailed description.

Step 3: Calculate the absorption and reflection properties of the coupled multi-unit structure using the mode matching method (see Supplementary Materials Note 3A). This step connects the impedance of individual units to the overall performance.

Optimization Process: By following the above steps, the absorption performance can be optimized using algorithms such as genetic algorithms. Instead of designing manually, these algorithms automatically enable the design of the mode density and Q-weighted mode density distribution based on the desired absorption spectrum and structural constraints (e.g., absorber geometry, number of resonators, number of layers, and mesh size).

After optimization, the performance of the output structure can be evaluated according to the performance Evaluation Metrics: R_{CT} (causality thickness ratio): By comparing the causality-constrained minimum thickness (calculated from the absorption spectrum) with the actual structural thickness, $R_{\mathrm{CT}} = L_{\min}/L$ is used to evaluate whether the absorber achieves optimal thickness. M_{BW} (bandwidth multiple): Calculated from the absorption spectrum, M_{BW} evaluates the broadband performance of the absorber. Larger M_{BW} values correspond to better broadband performance but also greater design challenges. The above

workflow is depicted as a flowchart in Fig. S8.

E. The comparison between juxtaposed structures and co-optimized designs.

For N structures with impedances $Z_n (n = 1, 2..N)$ and areas $S_n (n = 1, 2..N)$, the total impedance after juxtaposition is $Z_{\text{tot}} = \left(\sum_{n=1}^{N} {Z_n}^{-1} * S_n / \sum_{n=1}^{N} S_n \right)^{-1}$. As shown in Fig. S9(a), four 25mm-edge structures individually achieve quasi-perfect absorption in 200-400Hz, 400-800Hz, 800-1600Hz, and 1600-3200Hz bands. However, when juxtaposed, the net impedance becomes $Z_{\text{tot}} = \left(\sum_{n=1}^{4} {Z_n}^{-1}/4 \right)^{-1}$, leading to severe performance degradation (Fig. S9(b)). This stems from the effective impedance of each unit transforming to $Z'_n = Z_n \sum_{m=1}^{4} S_m / S_n = 4 * Z_n$, causing impedance mismatch. Thus, ultra-broadband absorption requires co-optimizing the entire spectrum. Fig. S14(b) demonstrates that despite moderate individual performance, collective tuning achieves 7-octave high-efficiency absorption. Furthermore, combining high-absorption juxtaposed components while accounting for area-dependent impedance effects has the potential to achieve efficient absorption. It should be noted, however, that meticulous consideration must be given to the coupling among components when employing this approach.

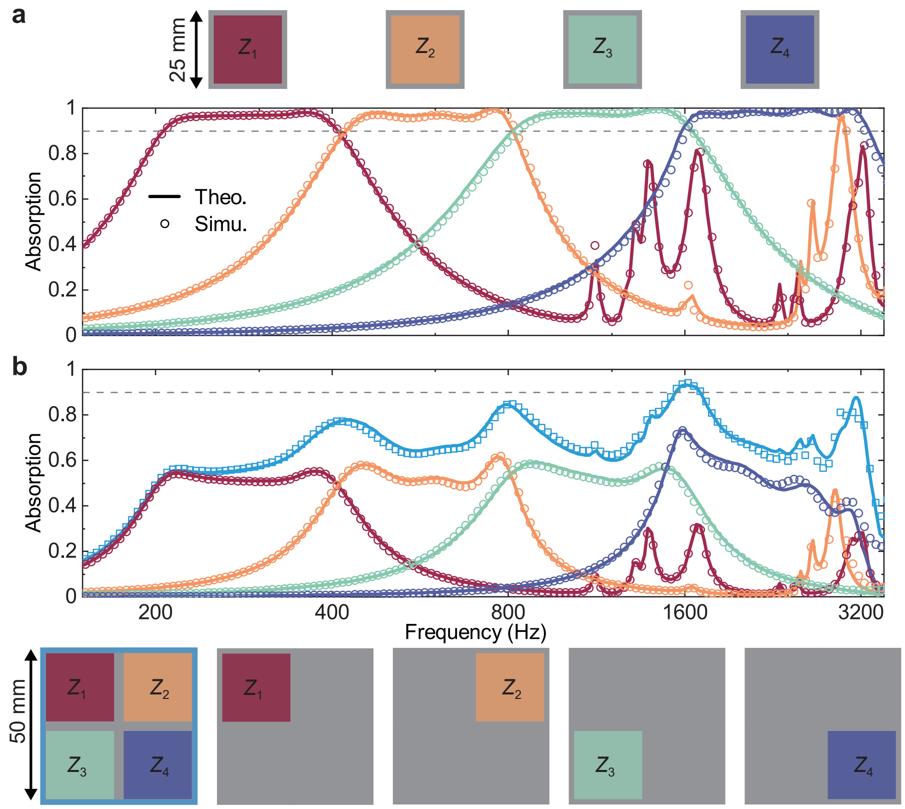

Fig. S9 | Demonstration of absorption effect by juxtaposing different structural units. **a**, Theoretical (curves) and simulated (circles) absorption performance of four distinct structures when individually placed. Insets show conceptual diagrams of the structures corresponding to the colored curves. **b**, Theoretical (curves) and simulated (circles) absorption performance of the four structures when juxtaposed (blue), aligned with the conceptual diagram in the blue frame. Theoretical (curves) and simulated (circles) absorption performance of individual structures after juxtaposition, matched with their respective colored structural conceptual diagrams.

Note 4: Detailed theory and comparison related to wire mesh.

A. Modulation of additional resistance on resonant modes.

By covering the structure with a thin layer of material (e.g., wire mesh (Fig. S10), with a thickness much less than the wavelength), the total acoustic impedance

$$Z_{\mathrm{t}} = Z + \Delta Z, \tag{S28}$$

where Z_{t} is the impedance of the structure and ΔZ is the additional impedance of the thin layer of material. Let the impedance $Z = R + iX$, from Methods section 'CMT for MRM'

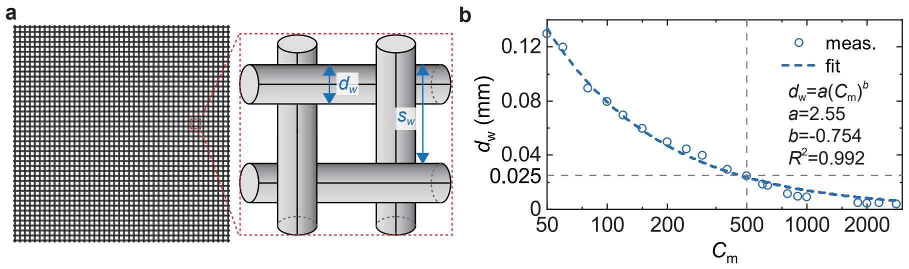

Fig. S10 | Schematic and parametric relationship of wire mesh. a, Schematic of wire mesh and an enlarged view in the right panel, where d_w is the wire diameter, and s_w is the wire spacing of wire mesh. **b**, Functional relationship between d_w and C_{m} fitted from measured data.

for a single resonant structure

$$R = \frac{\Gamma_1}{\gamma_1} X = \frac{\omega - \omega_1}{\gamma_1}. \tag{S29}$$

Assuming that the overall structure is still resonant after adding the additional impedance $\Delta Z = \Delta R + i\Delta X$, then

$$\begin{cases} R + \Delta R = \frac{\Gamma_1{}'}{\gamma_1{}'} \\ X + \Delta X = \frac{\omega - \omega_1{}'}{\gamma_1{}'} \end{cases}, \tag{S30}$$

which can be derived as

$$\begin{cases} \Delta R\left(\omega\right) = \frac{\Gamma_1{}'}{\gamma_1{}'} - \frac{\Gamma_1}{\gamma_1} \\ \Delta X\left(\omega\right) = \left(\frac{1}{\gamma_1{}'} - \frac{1}{\gamma_1}\right)\omega - \left(\frac{\omega_1{}'}{\gamma_1{}'} - \frac{\omega_1}{\gamma_1}\right) \end{cases}. \tag{S31}$$

This indicates that the additional impedance can only be a constant resistance and a linear function of the reactance. Meanwhile the total radiation loss, intrinsic loss and resonance frequency after adding the additional impedance can be expressed as $\Delta Z = C + i(D\omega + E)$

$$\begin{cases} \Gamma_1{}' = \frac{(\Gamma_1 + C\gamma_1)}{1 + D\gamma_1} \\ \gamma_1{}' = \frac{\gamma_1}{1 + D\gamma_1} \\ \omega_1{}' = \frac{\omega_1 - E\gamma_1}{1 + D\gamma_1} \end{cases} \overset{D=0, E=0}{\Rightarrow} \begin{cases} \Gamma_1{}' = \Gamma_1 + C\gamma_1 \\ \gamma_1{}' = \gamma_1 \\ \omega_1{}' = \omega_1 \end{cases}. \tag{S32}$$

It can be seen that the additional impedance of the individually added resistance ($\Delta Z = C$) only changes the intrinsic loss, and the decoupled tuning of the intrinsic loss and radiation loss, which can greatly improve the flexibility of response modulation of the MRM system. According to 'Impedance calculation of wire mesh' in the Methods, the wire mesh ($\Delta X \approx 0$) serves as an ideal material for individually added resistance (Fig. S2), avoiding extra phase

accumulation that would complicate impedance modulation. While for thicker materials it is necessary to calculate the total impedance with impedance transfer method, which increases the complexity of broadband modulation for impedance.

B. Comparison of the effect of different C_m and different materials on absorption. Wire mesh with different C_m exhibits distinct additional acoustic impedances due to variations in wire diameter. Optimal C_m selection enables dip-free high-efficiency absorption in the target frequency band while minimizing redundant absorption in non-target regions (Fig. S11).

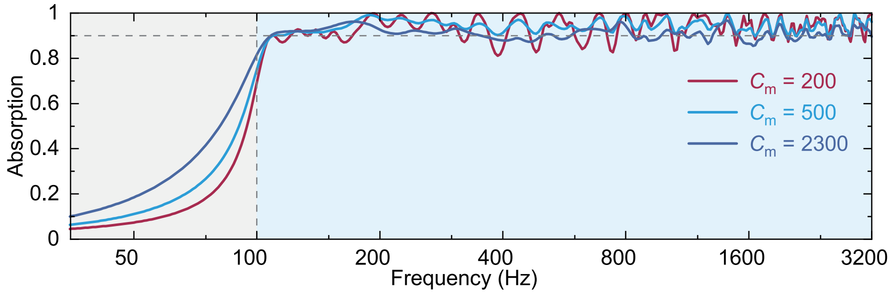

Fig. S11 | Comparison of absorption performance of absorbers with different C_m. The 200-mesh wire mesh ($r_a = 0.02$) provides insufficient resistance to suppress antiresonances, resulting in multiple absorption dips (red curve) within the target band (light blue region). The 2300-mesh wire mesh ($r_a = 0.21$) introduces excessive resistance, overbroadening resonance peaks and causing redundant absorption in non-target low-frequency bands (gray region,), which weakens performance (dark blue curve)in the target region. The 500-mesh configuration achieves an optimal trade-off, maintaining near-perfect absorption (blue curve) across the target band.

Taking the 500-mesh wire mesh as a reference, a sponge layer of equivalent thickness exhibits negligible acoustic modulation effects. To achieve comparable sound absorption performance, the sponge layer must be 220 times thicker than the wire mesh, resulting in increased absorber thickness (Fig. S12). Furthermore, compared to sponges, the wire mesh demonstrates superior resistance to airflow washout, enhanced mechanical robustness, broader temperature tolerance, and improved corrosion resistance, making it more competitive for practical applications.

C. Comparative performance of MMA with/without wire mesh.

The MMA without wire mesh exhibits weaker χ_{QMD} and Q^{-1}compared to its counterpart

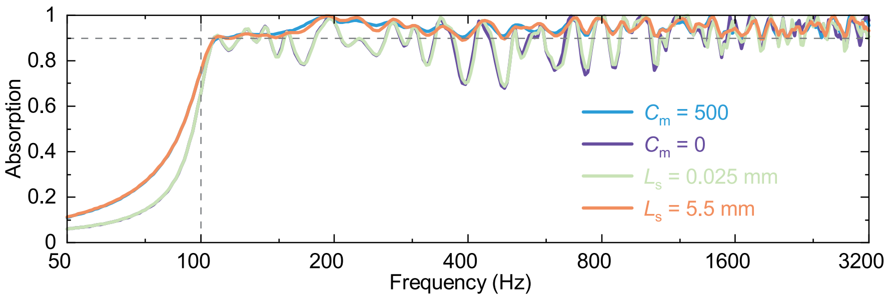

Fig. S12 | Comparison of sponge and wire mesh in modulating absorber performance. A sponge layer of the same thickness as the 500-mesh wire mesh ($L_s = 0.025$ mm, light green curve) exhibits nearly identical absorption performance to the bare structure ($C_m = 0$, purple curve). A 5.5 mm sponge layer (220× thicker than the wire mesh, orange curve) is required to achieve absorption comparable to the 500-mesh wire mesh (blue curve).

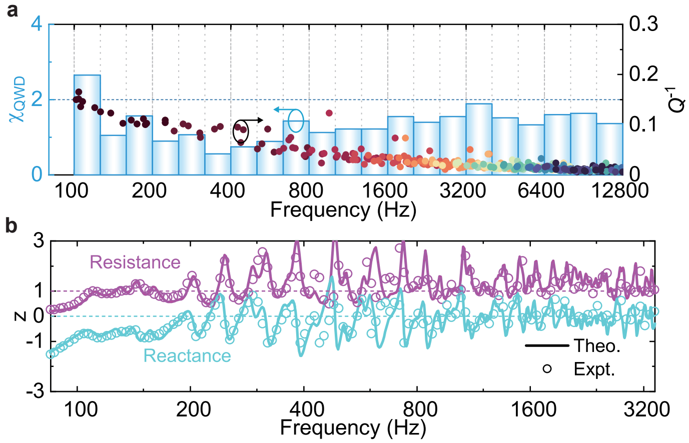

Fig. S13 | The Q^{-1} and χ_{QMD}, and acoustic impedance spectra of the MMA not covered with wire mesh. a, The χ_{QMD} and Q^{-1} of the MMA without wire mesh covering, which values significantly smaller than those in Fig. 3b. **b**, The acoustic impedance spectra of the MMA without wire mesh covering, both theoretical and experimental, exhibit significant oscillations when compared to Fig. 3d. The introduction of the wire mesh significantly improves the χ_{QMD} and effectively suppresses the resonance dispersion.

with wire mesh, accompanied by impedance spectral oscillations (Fig. S13). Although individual resonator units demonstrate better absorption characteristics, the MMA has many absorption dips(Fig. S14). The evolution of MMA absorption spectra aligns well with the theoretical predictions in Fig. S1. The reflection coefficient of MMA without wire mesh displays multiple reflection zeros in the upper half complex plane, with its phase spectrum

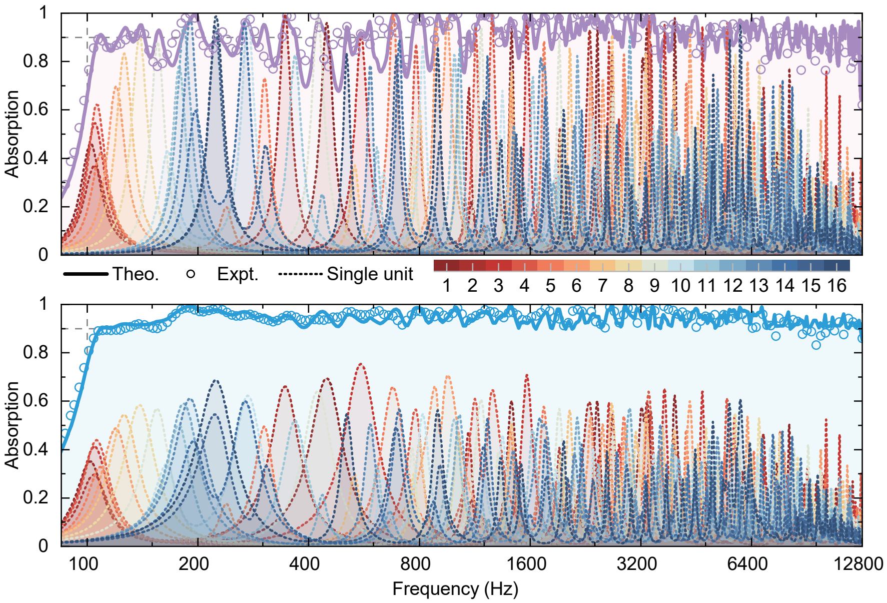

Fig. S14 | Comparison of the overall absorption performance of MMA. MMA without (top) and with (bottom) wire mesh and the absorption performance of individual units (dash lines). The lower panel corresponds to Fig. 3c.

showing multiple 2π phase-shifts (Fig. S15), which indicates the presence of redundant structural thickness in the absorber (see Note 6).

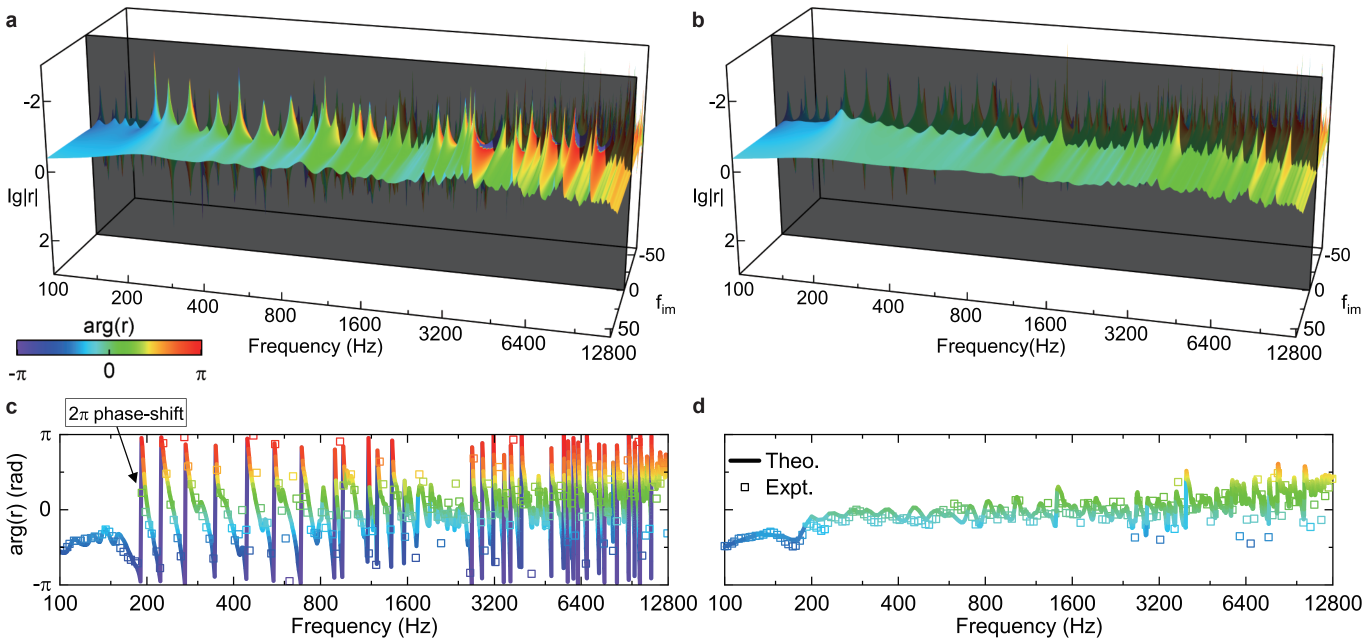

Fig. S15 | Variation of the characterization of the reflection coefficient by introducing wire mesh. a,b, Reflection coefficient complex frequency Riemann surface of the plane wave mode corresponding to the MMA without (**a**) and with (**b**) the wire mesh. In sharp contrast to the MMA without the wire mesh, the MMA with the wire mesh has no reflection zeros in the upper half-complex frequency plane. **c,d,** Phases of the reflection coefficients of the plane-wave modes corresponding to the MMA before without (**c**) and with (**d**) the wire mesh. Solid lines are theoretical values and squares are experimental values. The MMA covered with the wire mesh has the minimum phase shift frequency dependence across the target band.

Note 5: Oblique incidence absorption performance of MMA.

Due to the appropriate over-resistance characteristics (resistance between 1 and 2), the MMA achieves almost quasi-perfect absorption over a wide range of incidence angles from -60° to 60°(Fig. S16), and still maintains high absorption at larger angles (e.g., 75°).

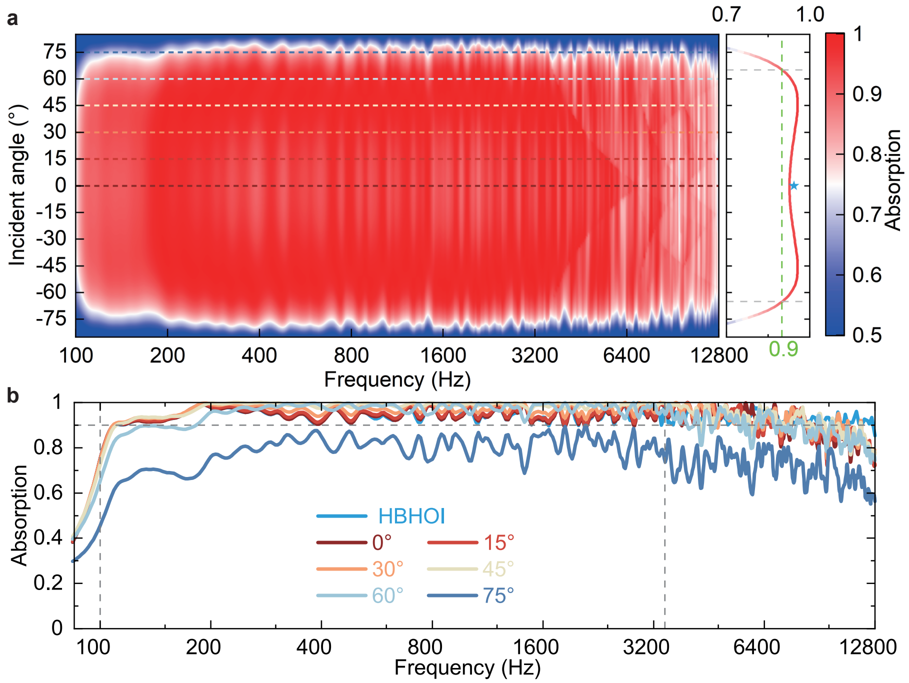

Fig. S16 | Absorption spectra of MMA at different angles of incidence. The right panel in **a** shows the average absorption coefficient in the target band. The calculation of the absorption coefficient takes into account periodic boundaries, and there are no multiple propagation modes of incidence. Therefore, there is a slight difference in high frequencies between the absorption spectra at vertical incidence (0°) and the absorption spectra in the main text, where hard boundaries are taken into account (HBHOI).

Note 6: Details related to the minimum thickness of the causal constraints.

A. Retrieve the minimum thickness from the absorption spectrum.

The minimum thickness L_{min} required by the causality constraint with respect to specific absorption spectrum can be calculated according to:

$$L_{\mathrm{min}} = \frac{\sigma}{4\pi^2} \left| \int_0^\infty \ln\left[1-\alpha\left(\lambda\right)\right] d\lambda \right|, \tag{S33}$$

where for acoustic waves, $\sigma = B_{\mathrm{eff}}/B_0$, and for electromagnetic waves, $\sigma = \varepsilon_0/\varepsilon_{\mathrm{eff}}$. Here, B_0 and ε_0 represent the modulus and permittivity of air (or another surrounding medium), respectively, while B_{eff} and $\varepsilon_{\mathrm{eff}}$ represent the equivalent modulus and permittivity of the absorber in the static regime ($\lambda \to \infty$). A smaller B_{eff} enables a reduced L_{min}, as derived from the relationship $B_{\mathrm{eff}}^{-1} = \varphi B_{\mathrm{a}}^{-1} + (1-\varphi)B_{\mathrm{s}}^{-1}$, where B_{a} is the bulk modulus of air in the static state, B_{s} is the bulk modulus of the solid structural framework, and φ represents the air volume fraction within the absorber. Given $B_{\mathrm{s}} \gg B_{\mathrm{a}}$, the effective modulus simplifies to $B_{\mathrm{eff}} \approx B_{\mathrm{a}}/\varphi$. To minimize B_{eff}, we maximized φ by adopting ultra-thin walls while ensuring structural stability, thereby achieving a smaller L_{min}. Additionally, L_{min} is governed by the absorption spectrum $\alpha(\lambda)$ across the entire bandwidth. By suppressing absorption in non-target regions (e.g., low-frequency wavelengths) and designing a steeply rising absorption curve at the target band's lower edge, redundant absorption is minimized, optimizing thickness utilization efficiency. For instance, compared to foam of identical thickness, our MMA delivers superior absorption in the target band and allocates most of its thickness to absorption contributions within this band (Fig. S18). By substituting the desired absorption spectrum into this equation, the corresponding minimum thickness L_{min} can be easily calculated. The actual structural thickness satisfies $L > L_{\mathrm{min}}$. In the main text, the parameter R_{CT} is defined as $R_{\mathrm{CT}} = L_{\mathrm{min}}/L$, where R_{CT} has an upper limit of 1. A larger R_{CT} indicates that the thickness of the absorber is closer to the minimum thickness corresponding to the given absorption spectrum.

B. Phase spectrum characteristics of reflection for achieving minimum thickness.

For a rigid-backed absorber in a causal linear time-invariant system, the thickness L and absorption spectrum $\alpha(\lambda)$ satisfy the identity:

$$L = \frac{1}{4\pi^2}\frac{B_{\mathrm{eff}}}{B_0} \left| \int_0^\infty \ln\left[1-\alpha(\lambda)\right]\, d\lambda \right| + \frac{1}{2\pi}\frac{B_{\mathrm{eff}}}{B_0} \sum_n \left|\mathrm{Im}(\lambda_{\mathrm{n}})\right| \tag{S34}$$

where λ_n are zeros of the reflection coefficient in the upper half-plane of the complex frequency plane. When zeros exist in the upper half-plane (e.g., Fig. 2d), the second term on the right-hand side of Eq. (S34) is greater than zero, indicating redundant thickness. In this case, the phase singularities of the reflection coefficient are distributed on both sides of the real axis (Fig. S17a), and the reflection coefficient phase spectrum exhibits a 2π phase shift (Fig. S17c). Conversely, when no zeros exist in the upper half-plane (e.g., Fig. 2d), the second term vanishes, corresponding to the minimum thickness required for the absorption spectrum $\alpha(\lambda)$. Here, the phase singularities lie on one side of the real axis (Fig. S17b), and the reflection coefficient phase demonstrates minimum-phase-shift frequency dependence characteristics (i.e., a phase shift less than 2π across the spectrum; see Fig. S17d). This relationship further leads to the inequality:

$$L \geq L_{\min} = \frac{1}{4\pi^2} \frac{B_{\text{eff}}}{B_0} \left| \int_0^\infty \ln\left[1 - \alpha(\lambda)\right] \, d\lambda \right| \tag{S35}$$

Equality in Eq. (S35) holds when no zeros exist in the upper half-plane.

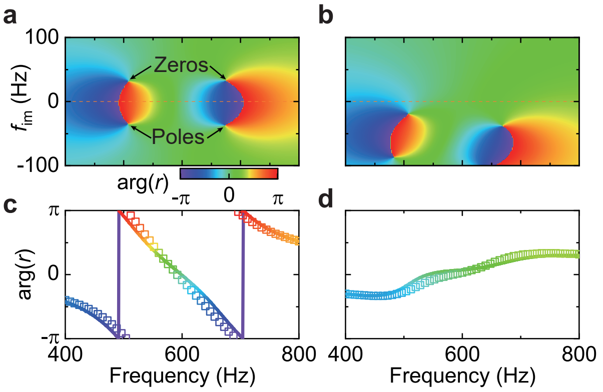

Fig. S17 | Phase Characteristics of the reflection coefficient for the structure in Fig. 2(c). a-b, Complex frequency-plane distributions of the reflection coefficient phase for bare (**a**) and wire mesh-covered (**b**) structures. **c-d**, Theoretical (solid lines) and simulated (squares) data of the reflection coefficient phase for bare (**c**) and wire mesh-covered (**d**) structures.

Note 7: Comparison of MMA and conventional absorber performance.

MMA has better absorption properties than conventional absorbers of the same thickness (melamine foam for example) in the important and tricky to manipulate low frequency region (Fig. S18). More importantly, MMA can realize extremely high tunable absorption spectra, for example, at 100Hz to 12800Hz with absorption coefficients 1,0,1,0.5,1,0,0.5 as the target of these 7 octaves, respectively, and by designing the Q-weighted mode density, the absorption performance can be obtained in accordance with the target (Fig. S19). This is a capability that is difficult to realize by conventional absorbers due to the absence of tunability.

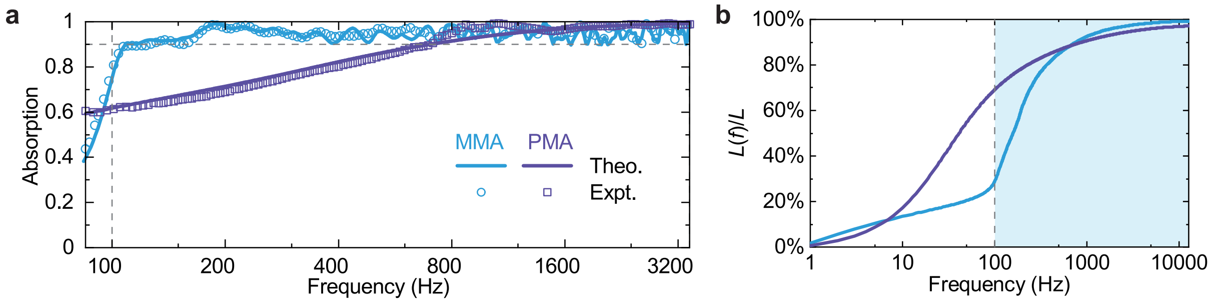

Fig. S18 | Comparison of the acoustic performance of MMA and porous material absorber (PMA e.g. melamine foam) of the same thickness in Fig. 3. a, Theoretical (curves) and experimental (squares) absorption spectra of MMA (purple) and PMA (blue). MMA exhibits a steeper rise in the initial band absorption spectrum compared to PMA, indicating less redundant absorption. Therefore, MMA demonstrates significantly better absorption performance than PMA in the low-frequency target regime (100-800 Hz) for the same sample thickness. **b**, The causal thickness proportion spectrum corresponding to absorption spectra of MMA (purple) and PMA (blue). Compared to the PMA, most of the thickness of the MMA is distributed in the target band (light blue region). Here, $L(f)$ is the thickness required for the absorption spectrum in the band 0 to f.

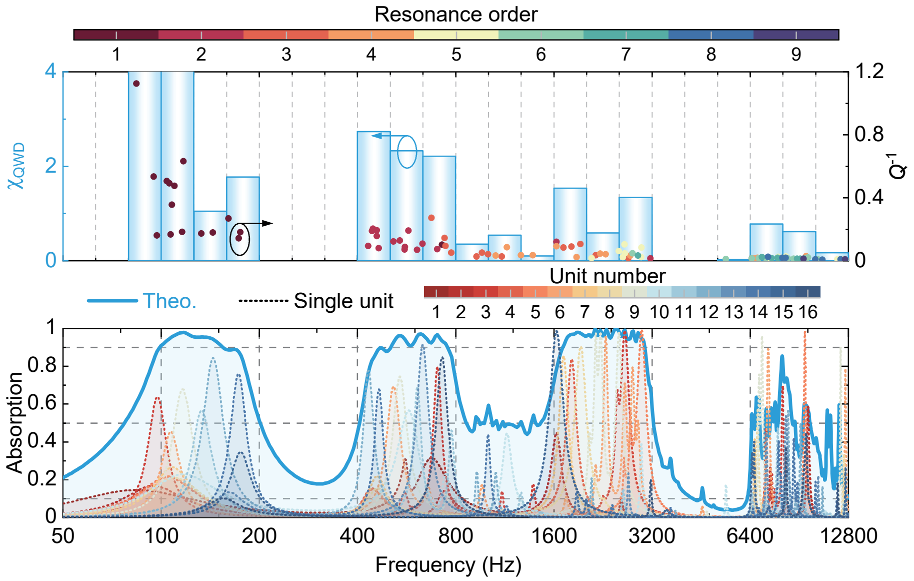

Fig. S19 | MMA with customizable absorption spectrum. Different absorption performance of MMA in various frequency bands can be achieved by assigning different χ_{QMD} values to each frequency band. This cannot be achieved with conventional absorbers.

Note 8: Comparison of different MMAs.

Compared with state-of-the-art MMAs (table S3), our MMA exhibits the greatest M_{BW} and R_{CT} values, while also exhibiting a nearly deep sub-wavelength thickness and excellent absorption. A comparison of operating bands for different MMAs is shown in Fig. S20, where our MMA demonstrates a significant bandwidth advantage over the existing broadband MMAs.

Reference	Operating band	quasi-perfect absorption band	M_{BW}	L	$L_{\min}$	R_{CT}	$L/\lambda_{\max}$	Average absorption
Our work	100-12800 Hz	109-12800 Hz	128 (117)	35.3 cm	35 cm	0.991	1/9.7	0.944 (0.941)
Ref. 8	4-20 kHz	N.A.	5	8.9 mm	8.2 mm	0.921	1/20	0.976 (0.903)
Ref. 16	345-3000 Hz	400-3000 Hz	8.7 (7.5)	10.86 cm	10.36 cm	0.954	1/9.2	N.A.
Ref. 7	320-6400 Hz	N.A.	20	100 mm	97.3 mm	0.973	1/11.4	0.93
Ref. 45	800-3200 Hz	N.A.	4	40 mm	38.6 mm	0.965	1/10.7	0.96
Ref. 37	870-3224 Hz	N.A.	3.7	39 mm	N.A.	N.A.	1/10.1	0.957
Ref. 46	420-10000 Hz	420-10000 Hz	23.8	0.2 m	N.A.	N.A.	1/4.1	0.991
Ref. 9	3-40 GHz	3-40 GHz	13.3	14.2 mm	13.5 mm	0.951	1/7	0.988
Ref. 39	2.1-9.1 GHz	2.1-9.1 GHz	4.3	13.3 mm	10.69 mm	0.804	1/10.7	0.983
Ref. 38	1.1-2 GHz	1.1-2 GHz	1.8	4 cm	2.8 cm	0.7	1/6.8	0.991
Ref. 47	5.2-18 GHz	5.2-18 GHz	3.5	1 cm	N.A.	N.A.	1/5.8	0.963
Ref. 48	0.3-1.8 μm	N.A.	6	330 nm	N.A.	N.A.	1/5.5	0.94
Ref. 40	300-2500 nm	N.A.	8.33	270 nm	N.A.	N.A.	1/9.3	0.85
Ref. 49	300-4500 nm	N.A.	15	620 nm	N.A.	N.A.	1/7.3	0.99
Ref. 50	2-40 GHz	2.3-40 GHz	20 (17.4)	30 mm	N.A.	N.A.	1/5	N.A.
Ref. 41	0.4-10 μm	N.A.	25	50 μm	N.A.	N.A.	5	0.99
Ref. 42	1200-2200 nm	1400-2000 nm	1.8 (1.4)	1730 nm	N.A.	N.A.	1/1.3	0.87

Table S3. Performance comparison of absorbers in different wave systems. Here, λ_{max} denotes the maximum wavelength of operating band, N.A. indicates not available in the cited work. The values in parentheses corresponding to M_{BW} and average absorption are data calculated from quasi-perfect absorption bandwidth calculations and experimental data, respectively. Larger M_{BW}, larger R_{CT}, smaller $L/\lambda_{\max}$, and larger average absorption denote better performance of the absorber. The data presented in the table are those presented in the citation and vary due to the recording conventions of different systems, but the quantities used to evaluate the performance are valid for all MMAs.

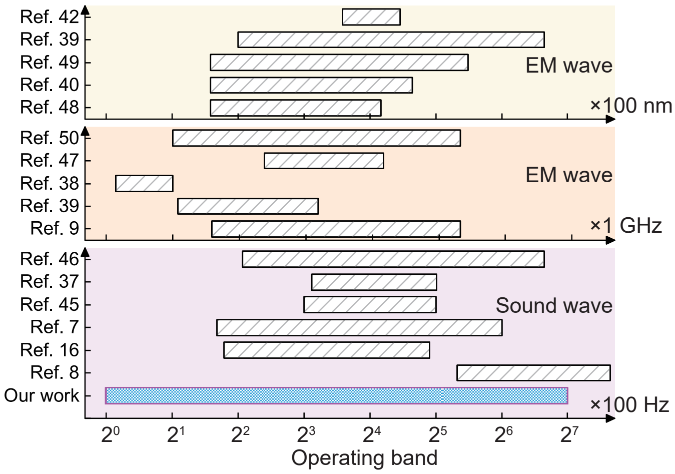

Fig. S20 | Comparison of the operating band of different MMAs. The detailed data is shown in table S3. Because of the different frequency bands studied and the recording conventions of different wave systems, the units corresponding to the horizontal coordinates corresponding to the three panels are different, but they are compared according to the same octave range.

Note 9: Details related to experimental theory.

A. Data analysis method.

The sound pressure at the measuring point with coordinate (x_h, y_h, z_h) in the standing wave tube is given by eq. S1 as

$$p\left(x_h, y_h, z_h\right) = \sum_{\lambda_{m,n}=\lambda_{1,1}}^{\lambda_{M,N}} \mathrm{X}_{\lambda_{m,n}}(x_h, y_h)(A^-_{m,n}e^{jk_{z,\lambda_{m,n}}z_h} + A^+_{m,n}e^{-jk_{z,\lambda_{m,n}}z_h}), \tag{S36}$$

where $\lambda_{m,n} = (n-1)\,M + m$ cuts off at $\lambda_{M,N}$. The above equation can be arranged into

$$p_h = \overrightarrow{Q^-}(h)\,\overrightarrow{A^-} + \overrightarrow{Q^+}(h)\,\overrightarrow{A^+}, \tag{S37}$$

where $p_h = p\left(x_h, y_h, z_h\right)$, and

$$\overrightarrow{Q^{\pm}}(h) = \begin{pmatrix} Q^{\pm}\left(h, \lambda_{-M,-N}\right) & Q^{\pm}\left(h, \lambda_{-M+1,-N}\right) & Q^{\pm}\left(h, \lambda_{m,n}\right) & Q^{\pm}\left(h, \lambda_{M,N}\right) \end{pmatrix}, \tag{S38}$$

With $Q^{\pm}\left(h, \lambda_{m,n}\right) = \mathrm{X}_{\lambda_{m,n}}(x_h, y_h)e^{\mp jk_{z,\lambda_{m,n}}z_h}$. A total of H measurement points, i.e., h is taken from 1 to H, and can be written in matrix form as

$$Q\,\overrightarrow{A} = \overrightarrow{P}, \tag{S39}$$

where $Q = \begin{pmatrix} Q^- & Q^+ \end{pmatrix}$, with

$$Q^{\pm} = \begin{pmatrix} \overrightarrow{Q^{\pm}(1)}^T & \overrightarrow{Q^{\pm}(2)}^T & \cdots & \overrightarrow{Q^{\pm}(h)}^T & \cdots & \overrightarrow{Q^{\pm}(H)}^T \end{pmatrix}^T, \tag{S40}$$

and

$$\vec{A} = \begin{pmatrix} \vec{A^-}^T & \vec{A^+}^T \end{pmatrix}^T. \tag{S41}$$

The absorption coefficients can be calculated by solving eq. S39 for the incident and reflected amplitudes of each order according to the section 'Mode matching method for calculating absorption'. The reflection coefficients for each mode can also be obtained as

$$r_{m,n} = \frac{A^+_{m,n}}{A^-_{m,n}}. \tag{S42}$$

The order m and n of the propagation modes of the T50 used in the measurement at $f_{\max}$ are truncated at 4, and the order (4,4) is also an evanescent wave mode, i.e., there are 15 propagation modes in total, and the coefficients of the propagation modes can be extracted by using the 32 microphones in section 'The experimental setup'. The location of the 32 microphones is shown in Table S4,and the distribution is schematized in Fig. S21.

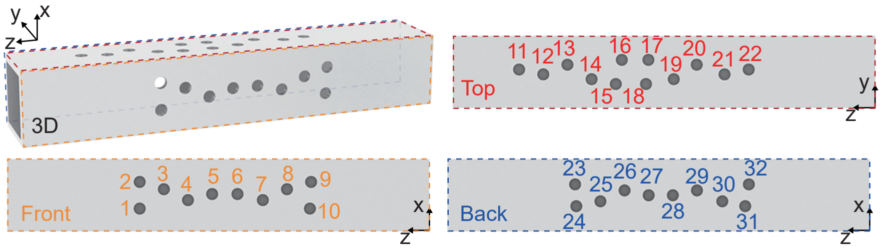

Fig. S21 | Distribution of the 32 microphones in the measurement section. The 4 panels are 3D view, front view, top view, and rear view. The circular diameter for each mounted microphone is 1/4 inch, and the detailed position data for the center of the circles are shown in table S4.

B. Dispersion relations for waveguide modes of each order in SWTs.

The dispersion relation of wave number in a wave system can be written as

$$k_{z,\lambda_{m,n}} = \sqrt{k_0^2 - k_{x,m}^2 - k_{y,n}^2}, \tag{S43}$$

where k_0 is the wave number in free space, $k_{z,\lambda_{m,n}}$, $k_{x,m}$ and $k_{y,n}$ are the z-direction, x-direction and y-direction components of the wave number of the waveguide mode of

Point number	1	2	3	4	5	6	7	8	9	10		
x axis (mm)	14	36	30	21	26	26	21	30	36	14		
y axis (mm)	0	0	0	0	0	0	0	0	0	0		
z axis (mm)	240	240	220	200	180	159	138	118	98	99		
Point number	11	12	13	14	15	16	17	18	19	20	21	22
x axis (mm)	50	50	50	50	50	50	50	50	50	50	50	50
y axis (mm)	27	23	31	19	15	35	35	15	19	31	23	27
z axis (mm)	295	275	255	235	215	210	188	190	167	148	125	105
Point number	23	24	25	26	27	28	29	30	31	32		
x axis (mm)	34	16	20	29	25	25	29	20	16	34		
y axis (mm)	50	50	50	50	50	50	50	50	50	50		
z axis (mm)	244	243	223	203	183	163	143	122	103	100		

Table S4. Specific data on the microphone mounting position for the measurement section.

the (m, n)-th order, respectively. For the acoustic field inside the SWT corresponding to the acoustic field with hard boundary conditions described in Note 3A, the wave vector components in the x-direction and y-direction can be represented as $k_{x,m} = m\pi/a, m = 0, 1, 2, \cdots, M$ and $k_{y,n} = n\pi/b; n = 0, 1, 2, \cdots, N$. Then the dispersion relation for the square SWT with side length l can be written as

$$k_{z,\lambda_{m,n}} = \sqrt{k_0^2 - \left(\frac{m\pi}{l}\right)^2 - \left(\frac{n\pi}{l}\right)^2}. \tag{S44}$$

It is further rewritten as

$$\frac{k_{z,\lambda_{m,n}} l}{\pi} = \sqrt{\left(\frac{k_0 l}{\pi}\right)^2 - (m^2 + n^2)}. \tag{S45}$$

Thereby the dispersion relations for the waveguide modes with different orders are shown in Fig. S22. According to the acoustic wave equation eq. S1, the modes corresponding to $k_{z,\lambda_{m,n}}$ for real numbers can propagate, while $k_{z,\lambda_{m,n}}$ for imaginary numbers the mode will dissipate quickly. And from the above equation, the $k_{z,\lambda_{m,n}}$ of $(m+1, n+1)$ and $(n+1, m+1)$ order are exactly the same, so these two modes will appear together, e.g., (1,2) and (2,1) in Fig. S22 are exactly coincident. The plane-wave mode corresponds to $(m, n) = (0, 0)$. When $k_0 l/\pi < 1$

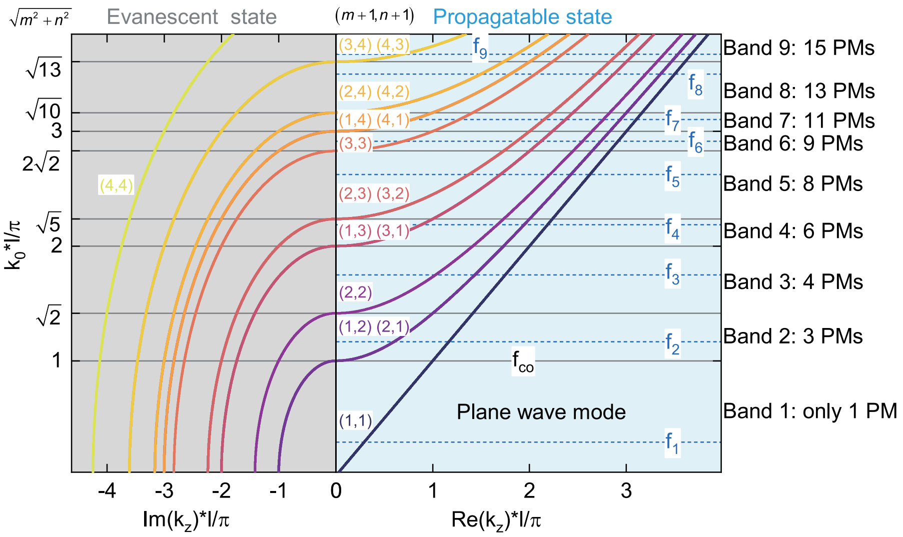

Fig. S22 | Dispersion curves for each order mode in the square SWT. The grey region is the evanescent state, corresponding to an imaginary k_z, while the green region is the propagatable state, corresponding to a real k_z. The colored solid lines denote the dispersion curves of different waveguide modes with orders corresponding to markers of the same color. The dark grey solid line divides the target band of Fig. 3 into 9 bands according to the different number of propagatable modes (PMs), with 1, 3, 4, 6, 8, 9, 11, 13, and 15 PMs, respectively. f_{co} denotes the cutoff frequency of only the plane wave, corresponding to $k_0 l/\pi = 1$. The green dashed lines correspond to the 9 frequency points selected in Fig. 3c and Fig. 4, each distributed in a different frequency band.

(i.e., $f < c/(2l)$), only plane-wave modes propagate in the waveguide, making $f_{\text{co}} = c/(2l)$ the cutoff frequency for exclusive plane-wave propagation. For the square waveguide with width $l = W = 5$ cm and sound speed $c_0 = 343.2$ m/s at room temperature, the cutoff frequency is $f_{\text{co}} = c_0/(2l) = 343.2/(2 \times 0.05) = 3432$ Hz. This rational value ensures a practical cross-sectional aspect ratio of 5 cm : 35.3 cm $\approx$ 1 : 7 for the absorber, facilitating sample fabrication. Simultaneously, the maximum number of propagating modes at the highest frequency (12800 Hz) is limited to 15, enabling accurate experimental measurements. Experimental results demonstrate that the constructed MMA exhibits superior broadband absorption performance for both plane-wave modes and higher-order modes (Fig. 4 and S23). Analysis of the nine modes present at f6 reveals that the majority of the incident energy is absorbed and a tiny portion is reflected after transformation between different modes, which

is the reason why the amplitude of the reflection coefficients of several propagatable modes in Fig. 4 is slightly larger than the amplitude of the incident coefficients. Additionally, due to the structural heterogeneity, the conversion between different modes also varies, which further contributes to the absorber's distinct absorption behaviors for the symmetric modes (m,n) and (n,m) (Fig. S24).

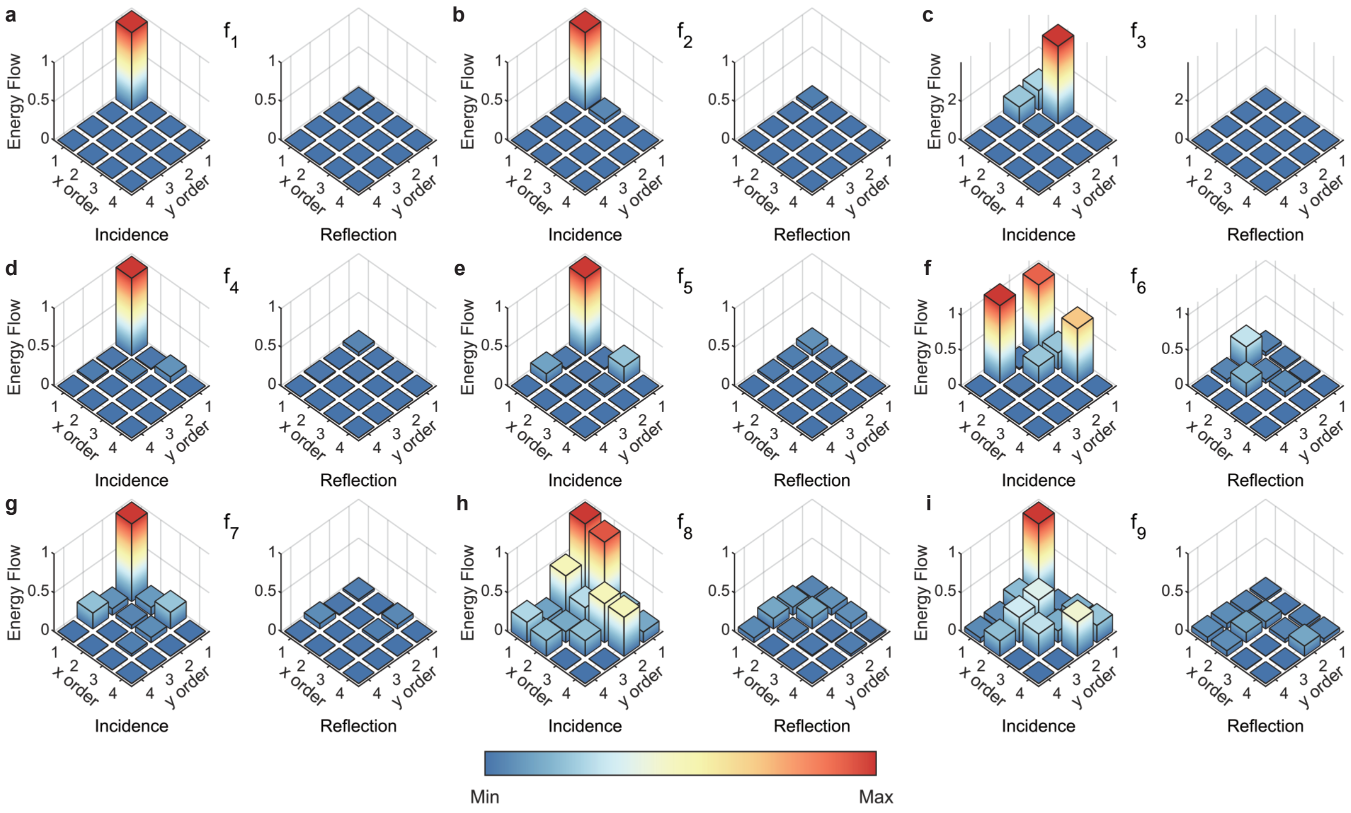

Fig. S23 | Energy flows of each order measured experimentally. a-i, Incident and reflected energy flows in the experiment corresponding to the frequency points f_1-f_9 with different numbers of propagation modes in Fig. 4c-k. The vast majority of the energy is absorbed, especially the dominant plane wave modes.

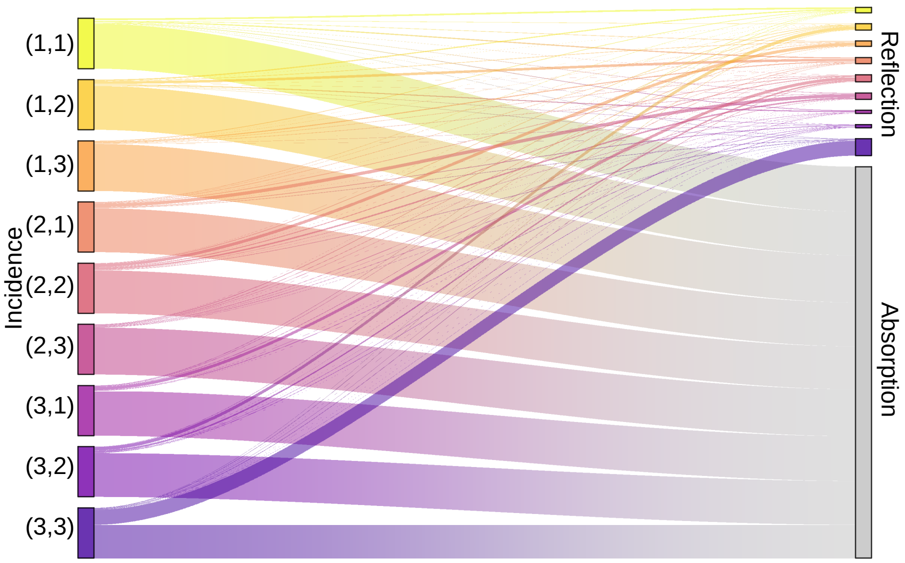

Fig. S24 | Transformation between propagatable modes. Energy transformation between modes of f_6 with 3*3 propagatable modes.
